# A Clinical Update and Radiologic Review of Pediatric Orbital and Ocular Tumors

**DOI:** 10.1155/2013/975908

**Published:** 2013-03-12

**Authors:** Ajay A. Rao, John H. Naheedy, James Y.-Y. Chen, Shira L. Robbins, Hema L. Ramkumar

**Affiliations:** ^1^Department of Radiology, University of California-San Diego, 200 West Arbor Drive, San Diego, CA 92103, USA; ^2^Department of Radiology, Rady Children's Hospital San Diego, San Diego, CA 92123, USA; ^3^Department of Radiology, San Diego VA Medical Center, La Jolla, CA 92161, USA; ^4^Department of Ophthalmology, Ratner Children's Eye Center, University of California-San Diego, San Diego, CA 92093, USA; ^5^Department of Ophthalmology, Shiley Eye Center, University of California-San Diego, San Diego, CA 92093, USA

## Abstract

While pediatric orbital tumors are most often managed in tertiary care centers, clinicians should be aware of the signs of intraocular and orbital neoplasms. In the pediatric population, a delay in diagnosis of orbital and intraocular lesions, even if benign, can lead to vision loss and deformity. Intraocular lesions reviewed are retinoblastoma, medulloepithelioma, and retinal astrocytic hamartoma. Orbital neoplasms reviewed are rhabdomyosarcoma, neuroblastoma metastases, optic pathway glioma, plexiform neurofibroma, leukemia, lymphoprolipherative disease, orbital inflammatory syndrome, dermoid and epidermoid inclusion cysts, and Langerhans' cell histiocytosis. Vascular lesions reviewed are infantile hemangioma and venous lymphatic malformation. In conjunction with clinical examination, high-resolution ophthalmic imaging and radiologic imaging play an important role in making a diagnosis and differentiating between benign and likely malignant processes. The radiologic imaging characteristics of these lesions will be discussed to facilitate prompt diagnosis and treatment. The current treatment modalities and management of tumors will also be reviewed.

## 1. Introduction

The wide varieties of rare intraocular and orbital neoplasms differ in presentation in the pediatric population when compared to these same lesions in adults. While most pediatric ophthalmic tumors are benign, they may have a significant impact on vision and may result in significant morbidity and mortality. We categorize these diseases according to etiologies as neoplastic and vascular. Some congenital tumors may present in the first year of life, while others typically present later in childhood. Clinical examination signs that should raise concern include leukocoria (white pupil), strabismus, restriction of ocular motility, asymmetric eye position within the orbit, decreased vision, high pressure in the eye, inflammation of the eyelids or conjunctiva, pseudohypopyon (inferior whitish layer in the anterior chamber of tumor cells), vitreous hemorrhage or inflammation, and an afferent pupillary defect. However, these are often late findings. Clinical presentation combined with the characteristic imaging features of the disease can narrow differential diagnoses. Imaging modalities most often used to evaluate these lesions include orbital ultrasonography (US), computed tomography (CT), and most importantly magnetic resonance imaging (MRI). Ophthalmic pathology is also critical to come to a diagnosis. In this paper, we review the epidemiology, clinical manifestations, current treatment modalities, and the imaging features that differentiate pediatric ocular and orbital lesions.

## 2. Intraocular Neoplasms

### 2.1. Retinoblastoma

Retinoblastoma is the most common intraocular malignancy in the pediatric population. The incidence of retinoblastoma is approximately one case per 15000–20000 live births, which amounts to 9000 new cases each year. There is no gender or racial predilection, and 90% of cases are diagnosed in patients under three years of age [1]. Biallelic mutations of the *RB1* tumor suppressor gene likely predispose retinal progenitor cells to tumor growth [2]. In the heritable form, the first mutation is constitutional, and the second is somatic, which causes bilateral disease in the majority of patients. In the nonheritable form, both allelic mutations are somatic, limiting disease to one eye with delayed presentation compared to those with the heritable form [3]. Very rarely, the heritable form of retinoblastoma can develop from primitive neuroectodermal cells in the suprasellar and pineal regions with resultant tri/tetralateral disease [4].

The most common initial sign of retinoblastoma is leukocoria, where the light emanating through the pupil is white light reflecting off the tumor instead of red light reflecting off the retina. Other signs of retinoblastoma may include decreased vision, strabismus, redness, pain, high pressure in the eye, cellulitic-like periocular inflammation, pseudohypopyon, and proptosis in late disease [3]. Young children rarely complain of changes in their vision making nonvisual presenting signs of retinoblastoma more commonly observed. Additionally, measuring vision in young children can be difficult to assess accurately. On fundoscopic exam, four patterns of growth can be seen. In the endophytic growth pattern, tumors are well visualized and extend from the deep retinal layer into the vitreous with vessels coursing into the mass. With exophytic growth, tumors extend from the retina outward into the subretinal space, with vessels coursing over the tumor. This growth pattern can be associated with retinal detachment, and total tumor extent may be underestimated. The third pattern is the most common, representing a mix of exophytic and endophytic growth. Extensive retinoblastoma can metastasize into the CNS and present with symptoms of meningitis.

The fourth pattern, diffuse infiltrating retinoblastoma, accounts for 1-2% of retinoblastomas and usually arises from the anterior retina in older children. This form usually does not form a mass or contain calcium. The resultant pseudohypopyon and floating vitreal tumor cells can be easily mistaken for uveitis or endophthalmitis and has thus been termed a masquerade disease. The absence of pain, conjunctival hyperemia, synechia (anatomic adhesions), cataract, and vitreous fibrosis suggests retinoblastoma rather than inflammation. Diffuse retinoblastomas start anteriorly and may enter the anterior chamber and fill the vitreous cavity but usually do not extend to the optic nerve. Spontaneous regression of tumor results in a shrunken, nonfunctioning globe [5]. 

The diagnosis of retinoblastoma is made noninvasively by exam under anesthesia with ophthalmoscopy, orbital ultrasound, and fluorescein angiography. Lumbar puncture and bone marrow biopsies are only performed in patients at high risk for having extraocular disease. Biopsies are not performed because of the risk of extraocular spread. Typically, neoplastic cells spread through the optic pathways via the optic nerve and can breach the pia to extend into the subarachnoid space. Direct extension through the choroid and sclera can occur with subsequent orbital extension. Finally, hematogenous metastases can spread to the lungs, bones, brain, and other viscera [3].

Historically, retinoblastoma was grouped into categories based on the Reese-Ellsworth classification system (groups I–V). The International Intraocular Retinoblastoma Classification System (IIRC) separates retinoblastomas into groups (A–E) based on prognosis, which is related to the extent of intraocular disease at diagnosis (Supplemental Table  1 will be available online at http://dx.doi.org/10.1155/2013/975908). More recently, the International Retinoblastoma Staging System (IRSS), proposed by a consensus of clinicians to separate retinoblastomas into stages based on management approach, has become popular [6]. Based on the IRSS classification, Stage 0 eyes can be treated conservatively. Stage I eyes are enucleated with complete histologic resection, and Stage II eyes are enucleated with residual microscopic tumor. Stage III eyes have regional extension, including local lymph nodes, while Stage IV patients have metastatic disease (hematogenous, CNS, multiple lesions) [6]. The Tumor, Node, and Metastasis (TNM) classification system is generally used for extension of retinoblastoma beyond the eye.

**Figure 1:**
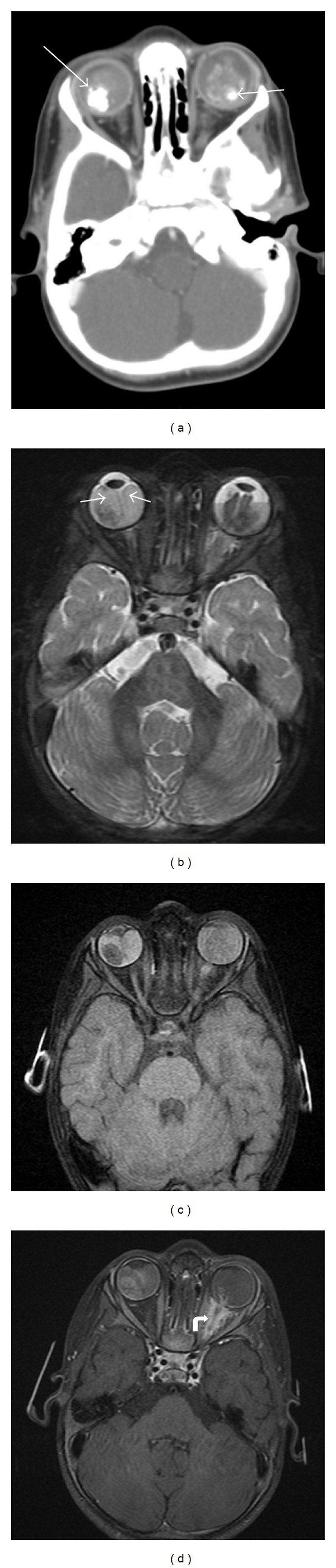
17 months old diagnosed with bilateral retinoblastoma requiring systemic chemotherapy. Axial contrast-enhanced CT through the orbits (a) demonstrates bilateral intraocular masses with coarse calcifications (arrows) and mild-to-moderate enhancement, diagnostic of retinoblastoma. MR axial T2-weighted image with fat-saturation (b) demonstrates curvilinear hypointensities in both globes, best visualized on the right, consistent with retinal detachment (arrows). Axial T1-weighted fat-saturated (c) and postcontrast T1-weighted fat-saturated (d) images show the bilateral intraocular masses with abnormal signal and enhancement in the intraconal left orbit along the optic nerve (curved arrow), which may represent a combination of postlaminar spread of tumor along the optic nerve and secondary perineuritis. This is not apparent on the CT scan.

Radiologic imaging can be used to help confirm the diagnosis and determine staging. The vast majority of nondiffuse type retinoblastomas appears nodular with calcifications, and presence of these calcifications distinguishes retinoblastoma from other intraocular lesions. CT evaluation of retinoblastoma (Figure 1(a)) typically demonstrates a hyperattenuating mass in the posterior globe with calcifications. On ultrasound, retinoblastomas are irregular masses that are more echogenic than vitreous and contain shadowing calcifications. Orbital ultrasound can detect calcifications in up to 95% of cases and can confirm the clinical diagnosis of retinoblastoma [7]. Conventional 1.5 T MR has not been considered as sensitive for detection of calcifications, but higher field-strength MR units and newer susceptibility-weighted sequences have increased its sensitivity. Galluzzi et al. found that when MR, ophthalmoscopy, and ultrasound were combined, none of the calcifications detected on CT were missed [7]. MR evaluation avoids the ionizing radiation of CT and is more sensitive for detection of extraocular extension of disease, perineural spread into the optic nerve (Figure 1), and involvement of the subarachnoid space; therefore, CT is no longer the preferred modality.

**Figure 2:**
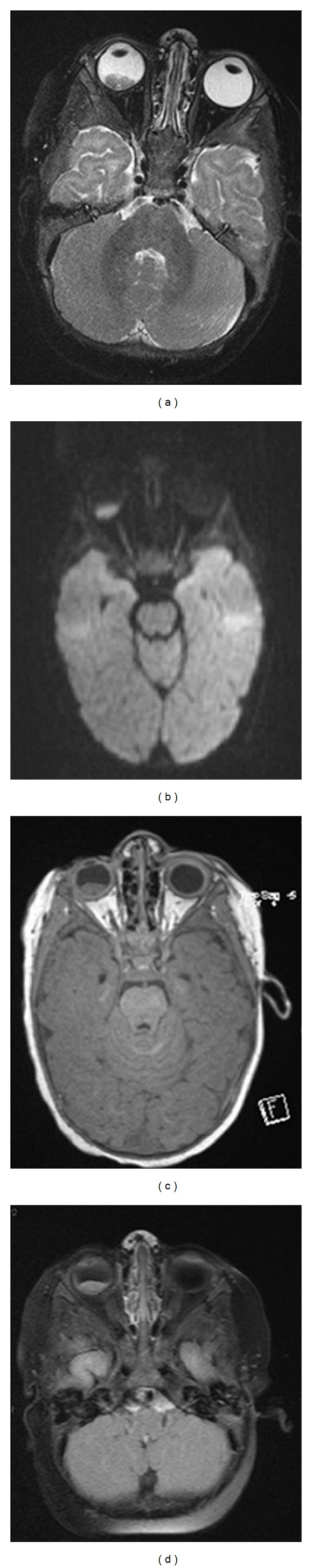
Axial T2-weighted image with fat saturation (a), diffusion-weighted image (b), T1-weighted image (c), and T1-weighted postcontrast image with fat saturation (d) demonstrate a unilateral mass in the right posterior globe that shows mild enhancement and no extraocular extension. This was diagnosed as retinoblastoma and the increased signal on diffusion imaging is compatible with the malignant and highly cellular nature of this disease.

MR is the study of choice for known retinoblastoma cases with clinical symptoms concerning for extraocular spread of disease and for followup evaluations. Retinoblastoma (Figure 2) follows signal intensity of gray matter with tumor hyperintense to vitreous on T1-weighted images (T1WI), hypointense compared to vitreous on T2-weighted images (T2WI), and enhances on postcontrast T1WI. The areas of enhancement also demonstrate high signal on diffusion-weighted images (DWI) and low signal on apparent diffusion coefficient (ADC) maps, which likely corresponds to cellular, viable tumor [8]. When contrast enhanced MR is performed, the minimal required sequences are as follows: T2WI through both orbits (2 mm or thinner); T2, precontrast T1 and postcontrast T1-weighted sequences through each diseased eye and optic nerve using axial and sagittal oblique planes (2 mm or thinner slices); and axial T2 and post-contrast T1-weighted sequences through the brain. Techniques to improve signal-to-noise ratio include using surface coils on a 1.5 T magnet or multichannel head coils on a 3 T magnet. The 3D steady state free precession sequences also provide high spatial resolution (slice thickness less than or equal to 1 mm) to aid in the detection of small lesions [9]. Fat saturation technique on postcontrast T1WI can help separate normal orbital fat from abnormal enhancement. If there is evidence of subarachnoid spread of disease, a complete spinal survey should be added to determine extent of spread through the neuroaxis.

Radiologic imaging is less sensitive and specific for determining the diffuse infiltrative pattern of disease, but this may show an anterior plaque without a discrete mass or calcifications. Ophthalmic imaging modalities, including ultrawide field photography, angiography, and spectral domain optic coherence tomography, aid in the diagnosis of this subtype of retinoblastoma. Enhancement is uniform with diffuse retinal thickening; however, micronodules may be visualized via US or MR with rare extension posteriorly through the choroid or into the optic nerve [10].

 When bilateral tumors are seen, retinoblastoma should be considered until proven otherwise. Other diseases that cause leukocoria can be differentiated from retinoblastoma based on imaging characteristics and clinical presentation. Coats' disease, persistent hyperplastic primary vitreous, and toxocara endophthalmitis lack calcifications. Retinopathy of prematurity can be bilateral and rarely calcified; however, a history of prematurity can be elicited, and the characteristic vascular patterns can be seen on exam. Lastly, retinal astrocytic hamartomas can calcify but are well circumscribed, lack hemorrhage or necrosis, and are associated with other findings related to tuberous sclerosis (TS) or neurofibromatosis 1 (NF1).

The treatment of retinoblastoma is complex and involves a multidisciplinary team, including pediatric oncologists, ophthalmologists, diagnostic and interventional radiologists, radiation oncologists, ocular pathologists, and geneticists. The available treatment options include enucleation (eye removal), chemoreduction, selective intraarterial and systemic chemotherapy, laser photocoagulation, focal cryotherapy, transpupillary thermotherapy, plaque brachytherapy, and external-beam radiation therapy (EBRT). Enucleation is the treatment of choice for advanced retinoblastoma with no potential for visual salvage. Historically, chemotherapy was reserved for extraocular or metastatic disease with adjuvant chemotherapy after enucleation. Discovery that patients who underwent EBRT are at an increased risk of developing secondary malignancies led to a paradigm shift in retinoblastoma treatment [11]. Now, systemic chemotherapy is used for chemoreduction, followed by laser coagulation, thermochemotherapy, cryotherapy, transpupillary thermotherapy, or plaque radiotherapy for treatments that spare the eye. 

Multiagent chemotherapy usually includes a combination of carboplatin and vincristine with or without etoposide. Studies have shown that for retinoblastomas in Reese-Ellsworth groups I–III, systemic chemotherapy in combination with local ophthalmic therapies (cryotherapy, laser photocoagulation, thermotherapy, and plaque radiation therapy) can avoid the need for enucleation or EBRT [11–13]. Some trials have demonstrated that treatment of large tumors with systemic chemotherapy in conjunction with localized therapies may be preferable, as they can provide vision and globe salvage with some benefits over enucleation [14]. Toxicities from chemotherapy include cytopenias (89%), neutropenic fever (28%), infection (9%), gastrointestinal symptoms, dehydration, and vincristine neurotoxicity (40%) [12]. 

More aggressive therapy is needed for Reese-Ellsworth groups IV and V, as advanced cases with diffuse vitreous seeding are extremely difficult to treat. Subconjunctival carboplatin and intravitreal carboplatin have been used in cases of diffuse vitreal seeding in Reese-Ellsworth group Vb eyes without evidence of seeding at 37 month followup [15]. This treatment also has improved disease control as has higher doses of carboplatin with etoposide or vincristine and combining vincristine, etoposide, and carboplatin. Many molecular factors play into the resistance of advanced retinoblastoma to chemotherapy and radiation. Cancer stem cells, expression of drug efflux protein pumps, mutations in protein kinases that alter cisplatin metabolism, and overexpression of HLA-G have been identified in enucleated retinoblastomas that have failed chemotherapy and EBRT [15–17].

Cryotherapy is effective for small (<3 mm apical thickness and <10 mm basal dimension) peripheral tumors, and a triple freeze-thaw technique is used with a tumor control rate of up to 90%. Multiple sessions of focal laser photocoagulation can be used alone for tumors less than four disc diameters. Thermotherapy with infrared radiation may also be used. When the aforementioned local treatments fail, brachytherapy is ideal for small, discrete, and accessible tumors. In a large study, plaques with radioactive ruthenium 106 or iodine 125 had a 79% tumor control rate at 5 years [18]. Proton beam radiation therapy, electron beam therapy, and intensity-modulated radiation therapy are modalities of EBRT used to reduce the dose of radiation used. Typically, a total dose of 40–45 Gy is delivered over 20–25 treatments over 4-5 weeks. The risk of secondary malignancies in retinoblastoma patients has been reported with some modalities of treatment, especially EBRT. 

Infusion of selective intraarterial chemotherapy (SIAC) is currently the new trend in retinoblastoma treatment. SIAC has been used for the primary treatment of unilateral and bilateral retinoblastoma in IIRC groups C and D with reported cures after one and two cycles of chemotherapy without major adverse events [18, 19]. In patients with advanced retinoblastoma (Reese-Ellsworth group 5a and b) that failed prior intravenous chemotherapy, SIAC with simultaneous carboplatin, topotecan, and melphalan has resulted in eye-sparing treatment in 75% of patients at 24 months followup. Recurrence rates were 35%, necessitating adjuvant local treatment; however, patients were all alive without evidence of metastatic disease in followup. Several patients had a decrease in the electroretinogram amplitude with treatment, likely secondary to chemotherapy, but retinal hemorrhages and optic nerve pallor were not noted in 14 month followup [18–20]. Central and partial retinal artery occlusion and choroidal atrophy has been reported in patients, but no cerebral ischemic events have been reported in studies [21, 22]. 

It has recently been reported that SIAC exposes patients to a significant amount of radiation [23] during each SIAC procedure, with one group measuring 191.73 mGy for the treated eye and 35.33 mGy for the fellow eye using a method that may have underestimated the total ocular radiation received [24]. Following a modified protocol for IAC with shorter fluoroscopy times and no subtraction angiography, radiation exposure can be reduced to 5.55 mGy in the treated eye and 1.68 mGy in the fellow eye [23] using the as low as reasonably achievable (ALARA) principle of radiation safety. The incidence of secondary cancers in patients who have received SIAC has not yet been reported, but this data should be followed and compared to the complications of EBRT. Currently, SIAC is considered an attractive option for both primary and secondary treatment of unilateral and bilateral retinoblastomas.

The future of ocular drug delivery now is focused on localized sustainable drug delivery directly in the eye. Transscleral depot reservoirs, liposomes, and nanoparticles are being investigated in preclinical models and may play a role in the future of treatment for intraocular malignancies like retinoblastoma. These treatments are being studied extensively for other retinal diseases with possible future applications in intraocular tumor treatment.

### 2.2. Medulloepithelioma

Medulloepithelioma is a rare congenital neuroepithelial tumor that arises from the nonpigmented ciliary epithelium of the ciliary body. Rarely, it can arise from the retina and optic nerve. It is slow growing and characterized as teratoid or nonteratoid and malignant or benign, based on histologic appearance. Population-based information on incidence is not available; however, most cases are found within the first decade of life and rarely late in adulthood [25]. This rare tumor is commonly misdiagnosed and treated as glaucoma and uveitis. Despite its slow growth, medulloepitheliomas can cause multiple secondary complications including secondary neovascular glaucoma, lens notching and subluxation, and neoplastic cyclitic membranes [26]. 

Reported medulloepithelioma cases describe a rapidly expanding ciliary body mass that masqueraded as chronic uveitis, cataract, and uncontrolled secondary glaucoma [27]. Clinically, it may appear as an amelanotic or lightly pigmented cystic mass in the ciliary body with erosion into the anterior chamber and iris root. On slit lamp exam, medulloepitheliomas are irregularly shaped with smooth surfaces and gray-pink color with conjunctival injection, sentinel episcleral vessels, intense anterior uveitis with fibrinous reaction, iris deposits, corneal stromal haze, and ectropion uvea. Decreased vision can arise from lens subluxation and cataract formation. If complicated by neovascular glaucoma, these patients may need aggressive surgical and medical management. Small tumors may be hidden by the iris, and larger tumors appear smooth and gray to pink in color. The presence of cysts along with tumor is highly suggestive of a medulloepithelioma [25].

**Figure 3:**
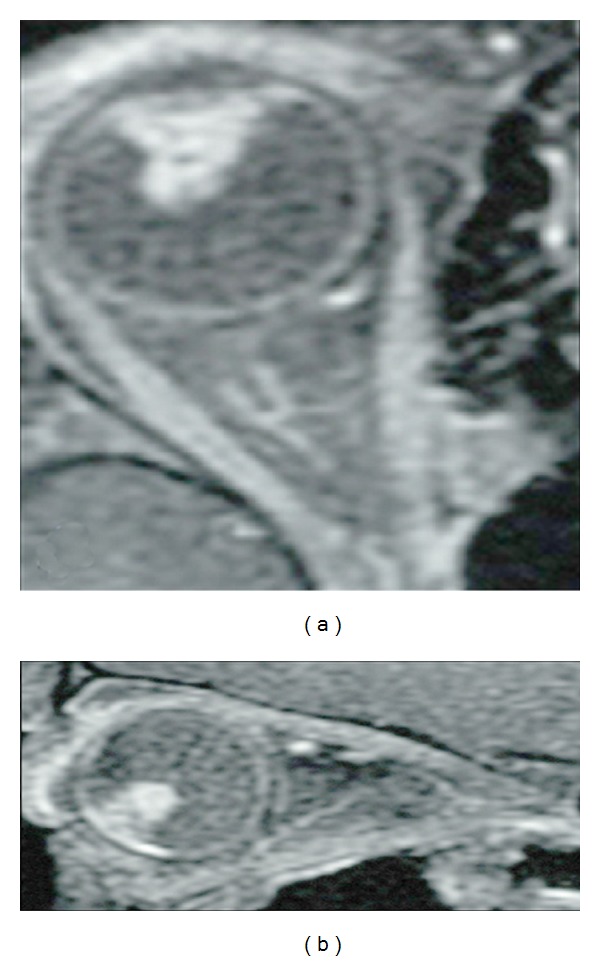
Axial (a) and sagittal (b) T1-weighted postcontrast images with fat saturation show an irregular, enhancing mass arising from the nonpigmented epithelium of the ciliary body. The tumor is confined to the globe. *Case courtesy of Dr. M. Mafee, UCSD Department of Radiology*.

Imaging can assist with determining extent of disease, and presence of extraocular spread of tumor with all imaging modalities typically demonstrates a solid mass with multiple characteristic cystic collections. Ultrasound biomicroscopy can be very useful in distinguishing anterior segment masses by providing high resolution images [28]. On B-mode ultrasound, the solid portion usually appears echogenic with irregular to ovoid shape. CT typically demonstrates a solid mass arising from the ciliary body with high sensitivity for detection of dystrophic calcifications that can be seen in up to 30% of the teratoid medulloepitheliomas [5]. Large tumors with calcifications that cannot be localized to the ciliary body may mimic a retinoblastoma in patients younger than 6 years of age [25]. On MRI, medulloepitheliomas demonstrate moderate hyperintense signal on T1WI, hypointensity on T2 images, and demonstrate moderate to marked enhancement (Figure 3) [4, 10].

Once the diagnosis is favored based on location, clinical appearance, imaging, staging, and size of the tumor are evaluated. Most medulloepitheliomas are cytologically malignant, but distant metastases are rare. Nodal metastases in the neck can occur with a predilection for the lymph nodes in the parotid gland. For small tumors, local excision via iridocyclectomy in conjunction with radiotherapy has been done with varying success [25, 28]. These cases often relapse, requiring secondary enucleation for local tumor recurrence or ocular inflammation and discomfort. Malignant medulloepitheliomas have been treated with iodine-125 plaque brachytherapy in combination with surgical excision. Enucleation, however, is considered the standard therapy once the diagnosis has been made [24, 29]. If the tumor extends beyond the globe, exenteration may be necessary. If medulloepithelioma is metastatic or involving other areas of the brain, a combination of chemotherapy and brachytherapy is initiated. Neoadjuvant chemotherapy includes combination ifosfamide, carboplatin, and etoposide. Multiple modalities of brachytherapy have been reported, including hyperfractionated radiotherapy of the craniospinal axis followed by a boost to the tumor site [30] and consolidating intrathecal brachytherapy using Yttrium 90 tagged with DOTA0-D-Phe1-Tyr3-octreotide (DOTATOC).

Though typically arising from the ciliary body, medulloepitheliomas have on rare occasion been reported arising from the optic nerve [31]. Most such cases have been malignant tumors. The clinical presentation includes unilateral proptosis, swollen eyelids, restricted ocular movements, pain, and poor visual acuity. After initial treatment with surgical excision, these cases may also relapse in the orbit, necessitating enucleation and exenteration. Patients are treated with aggressive chemotherapy with autologous bone marrow transplantation and radiotherapy. One-third of patients die from direct intracranial spread or CNS metastasis.

### 2.3. Retinal Astrocytic Hamartoma

Retinal astrocytic hamartomas are glial tumors of the retinal nerve fiber layer that arise from retinal astrocytes. They are cream-white, elevated, well-circumscribed lesions that may be multiple or solitary. There are two types of retinal astrocytic hamartomas: small, smooth, and flat noncalcified tumors that appear to be thickening of the nerve fiber layer or “mulberry” lesions, which are nodular large yellow-white calcified lesions. They can be found incidentally on exam, often in conjunction with tuberous sclerosis (TS) and rarely neurofibromatosis [32]. The incidence of tuberous sclerosis is approximately 1 in 10,000 persons, and about half of the people with TS have an astrocytic hamartoma. If detected, work-up for TS should be performed, especially in patients with characteristic skin lesions and a history of seizures or mental retardation. B-scan ultrasound, fluorescein angiography, and MR can all aid in making the diagnosis, but imaging is not routinely used for the detection or characterization of retinal astrocytic hamartomas, as clinical diagnosis is usually sufficient. Spectral domain optical coherence tomography can be used by ophthalmologists to evaluate the extent of the tumor and evaluate for macular edema or subretinal fluid. 

Usually, these lesions create no clinical symptoms, and treatment is rarely required. They can spontaneously resolve [33] but may become symptomatic by enlarging leaking, generating macular edema, accumulating lipid exudates, and forming serous retinal detachments [31, 33]. Additionally, elevated intraocular pressure and glaucoma have been reported in association with retinal astrocytic hamartomas. Vitreous seeding is a complication associated with inflammation and hemorrhage, which is managed with vitrectomy [34]. In some cases, spontaneous exudative hamartomas resolve after four weeks. If macular edema persists after 6 weeks; however, treatment is indicated, as delayed resorption of subretinal fluid can cause permanent visual impairment especially in young children. 

Argon laser photocoagulation has been reported to cause visual stabilization, but choroidal neovascularization is a reported complication. Photodynamic therapy is effective in resorption of subretinal fluid in these cases [35]. 

## 3. Orbital Neoplasms

### 3.1. Rhabdomyosarcoma

Rhabdomyosarcoma of the orbit represents the most common orbital malignancy in childhood, accounting for 10% of all rhabdomyosarcoma cases with mean age of 6–8 years of age [35, 36]. Children with the following rare inherited diseases Li-Fraumeni Syndrome, Beckwith-Widemann Syndrome, Neurofibromatosis type 2, Noonan Syndrome, and Multiple Endocrine Neoplasia type 2a Syndrome have an increased risk of this tumor. Rhabdomyosarcoma is a soft-tissue sarcoma with two major subtypes in the orbit: the more aggressive, less common alveolar type and the less aggressive, more common embryonal type (89%) [36]. Orbital rhabdomyosarcoma presents as a rapidly growing, painless mass that leads to proptosis, most commonly in the superomedial quadrant of the orbit for embryonal type and inferiorly for the alveolar type [37]. Anterior tumor involvement can involve the levator palpebrae superioris and lead to eyelid edema, hemorrhage, pain, isolated blepharoptosis, and chemosis [38]. Posterior involvement can cause vision changes when the optic nerve is compressed [36]. The rapid growth and aggressive nature of the tumor frequently result in invasion of the adjacent bone and soft tissue; however, local lymph node metastases and intracranial invasion are relatively uncommon [35, 36]. Hematogenous metastases most often spread to the lungs and bones [39]. Evaluation for metastatic disease may include imaging of the orbits and neck with CT/MRI, CT of the chest, lumbar puncture, bone scan, bilateral bone marrow aspiration, and core biopsies of the iliac crests with primary diagnosis made through open biopsy of the primary tumor [36]. Staging currently combines TNM staging and the clinical grouping classification for rhabdomyosarcoma.

**Figure 4:**
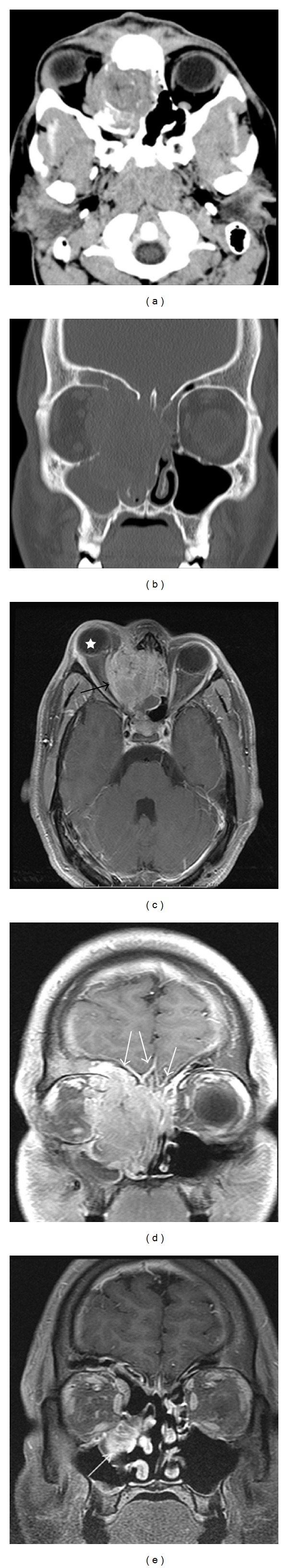
18-year-old male presenting with right sided proptosis and history of choroid plexus papilloma and seizures. Axial CT soft-tissue window (a) shows a soft-tissue mass centered in the right ethmoid sinus with bony destruction and invasion into the right orbit and left ethmoid sinus. Coronal CT image using bone windows (b) clearly demonstrates the osseous destruction and invasion of the right medial orbital wall, bilateral ethmoid sinuses, right frontal sinus, both nasal cavities, turbinates, and nasal septum. Postcontrast axial (c) and coronal (d) T1-weighted images with fat saturation better demonstrate the enhancing soft-tissue mass and its extension. The mass invades the medial orbit, displacing the right medial rectus laterally (black arrow), and causes proptosis (star). On coronal, the mass is again seen invading the adjacent sinuses and obstructing the nasal passage; however, unlike CT, abnormal enhancement is seen of the frontal dura (white arrows). The dural thickening and enhancement are compatible with direct tumor invasion. These findings were highly concerning for rhabdomyosarcoma. On postchemotherapy followup imaging (e), the tumor has significantly decreased in size with decreased mass effect on the orbit and adjacent structures. The previously seen dural enhancement has been resolved; however, some residual tumor remains in the paranasal sinus inferomedial to the orbit (white arrow).

On imaging, orbital CT and MRI are both helpful in evaluating extent of disease and are often complimentary. Orbital rhabdomyosarcoma appears isoattenuating to muscle on CT and is usually seen as a moderate to markedly enhancing extraconal, homogeneous, and well-circumscribed mass with calcifications only occurring with bony destruction (Figures 4(a) and 4(b)). Other common features include eyelid thickening and bone thinning/destruction, which can be seen in up to 40% of patients. Uncommon findings include necrosis, hemorrhage, and cavitation with ring enhancement [37]. CT of the chest may assess for pulmonary metastases in patients with rhabdomyosarcoma [40]. In contrast to CT, MRI provides excellent spatial resolution, superior soft-tissue contrast, and lacks radiation; however, evaluation of the bones is not as sensitive as CT [36, 39]. On MR imaging, tumor is isointense to muscle on T1WI, hyperintense to muscle on T2WI, and demonstrates moderate to marked enhancement postcontrast. The mass can distort/displace the globe and extraocular muscles but rarely invades these structures. Invasion intracranially or into the adjacent paranasal sinuses (Figures 4(c), 4(d), and 4(e)) is best depicted on comparison of pre- and postcontrast T1WI [37].

Nuclear medicine bone scintigraphy is currently used as part of the work-up for children with rhabdomyosarcoma and is most accurate in detecting osteoblastic metastases. Some studies have shown that PET-CT may be better at detecting bone and lymph node metastases than conventional anatomic imaging (CT, US) [41–43]. Additionally, two recent trials have compared whole body MRI (WBMRI) to conventional imaging and PET-CT for the evaluation of distant metastases in the pediatric population. Kumar et al. studied 26 patients with metastatic small cell neoplasms including rhabdomyosarcoma and found WBMRI to have a sensitivity of 97.5% and specificity of 99.4%, while PET-CT had a sensitivity of 90% and specificity of 100% [44]. Krohmer et al. found WBMRI to have a sensitivity of 96% in 24 patients with metastatic lymphoma or sarcoma with 190 lesions detected by MRI compared to 155 by PET-CT [45]. Newer diffusion-weighted techniques have shown utility in improving the diagnostic accuracy of WBMRI in patients with lymphoma, but this technique has not been applied to patients with rhabdomyosarcoma [46]. Whether PET-CT and WBMRI should be used routinely in staging, or followup remains unclear without a large prospective clinical trial specifically aimed at rhabdomyosarcoma; however, it can be considered in patients with increased risk of distant metastases.

The treatment of orbital rhabdomyosarcoma has changed drastically over the last 20 years from primary orbital exenteration to a more conservative approach combining systemic chemotherapy and radiation. Depending on the extent of tumor involvement, treatment may also involve surgical debulking. Patients routinely receive chemotherapy with vincristine and d-actinomycin [47]. Patients with a higher risk of relapse based on the Intergroup Rhabdomyosarcoma Study groups III and IV studies also receive cyclophosphamide. If complete surgical resection is confirmed by pathology, radiation can be withheld. More recent studies have shown that local control can be achieved with reduced dose radiotherapy (45 Gy), with a cumulative incidence of local failure of only 14% [48]. Orbital rhabdomyosarcomas have a 10-year survival rate of 87%. Recurrence is developed in 17% of patients at a median time of 18 months with local relapse rate of 92% and distant metastases rate of 8%. Complete remission was achieved in 96% of patients. Radiation is very effective in preventing and controlling local recurrence; however, overall survival was not affected by radiotherapy treatment [49]. The sequelae of radiation treatment in orbital rhabdomyosarcoma patients include, in order of frequency, reduced visual acuity, cataract, ptosis, orbital hypoplasia, dry eye, painful eye, retinal damage, maxillary hypoplasia, and corneal ulcers. Therefore, radiotherapy should be used sparingly and can be avoided in a subset of patients.

### 3.2. Neuroblastoma Metastases

Neuroblastoma is the most frequent extracranial solid tumor of childhood and arises from neural crest cells. The prevalence based on the SEER registry from 1973 to 2002 is 0.9 per 100,000 persons. The median age of diagnosis was one year, with 42% of patients presenting at less than one year of age [50]. It is the most common primary childhood cancer to metastasize to the orbits [51]. The mean age at diagnosis of patients with orbital neuroblastoma metastases is approximately two years of age. Twenty percent of all cases have orbital involvement, which can be the primary manifestation of the tumor [51–53].

The most common clinical presentation of orbital neuroblastoma metastases in patients less than two years old is unilateral or bilateral proptosis and periorbital or eyelid ecchymosis (raccoon eyes). Less common clinical findings include periorbital swelling, hemorrhage, strabismus, restricted ocular motility, ptosis, atrophy of the optic head, and ocular mobility disturbance [53]. Opsoclonus, characterized by rapid, multidirectional saccadic eye movements, is a paraneoplastic syndrome that is associated with neuroblastoma but not necessarily orbital involvement. This is generally a good prognostic factor, but neurologic deficits may remain. Primary thoracic neuroblastoma involving the sympathetic chain may present with Horner's syndrome (small pupil and ptotic eyelid). 

**Figure 5:**
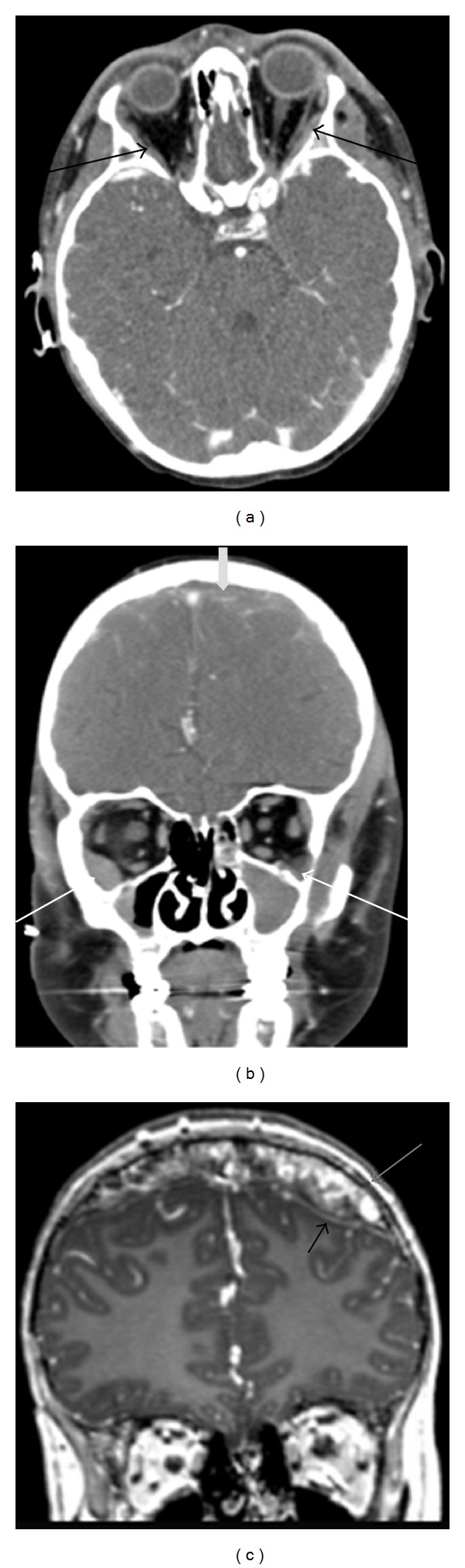
7 years old with history of treated neuroblastoma now with metastatic disease. Axial (a) and coronal (b) postcontrast CT images show ill-defined, slightly hyperattenuating soft-tissue masses along the inferior/lateral periorbital bones (black arrows and thin white arrows) and dural surfaces (thick grey arrow). MR coronal T1-weighted postcontrast image (c) also shows the diffuse, enhancing soft-tissue mass (grey arrow) extending along the dural surfaces (black arrow).

Like Langerhans cell histiocytosis, orbital neuroblastoma metastases tend to occur in the posterolateral orbital wall with CT and MRI providing better diagnostic information over ultrasound. On CT (Figures 5(a) and 5(b)), metastases can appear as either circumscribed or ill defined, are increased in attenuation compared to muscle, can contain small calcifications, and can invade adjacent structures including intracranially [53]. On MR, neuroblastoma metastases appear low signal on T1WI, heterogeneous on T2WI due to hemorrhage or necrosis, and heterogeneously enhance postcontrast [53]. Tumor extension intracranially and into the adjacent soft tissues may be seen to better advantage on MR over CT (Figure 5(c)). When multiple metastatic lesions are suspected, radioiodinated metaiodobenzylguanidine (MIBG) scintigraphy can be used for diagnosis, staging, and monitoring therapy with high sensitivity and specificity [54]. More recently, PET/CT has been shown to successfully stage and monitor disease with improved spatial resolution, improved detection of smaller lesions, and has the ability to provide anatomic detail for surgical planning [55].

Urine catecholamines have high sensitivity and specificity for neuroblastoma. Treatment of metastatic neuroblastoma is stratified based on clinical features (age at presentation, staging) and specific tumor biological markers that include histopathological analyses, chromosomal abnormalities, and quantification of expression of the *MYCN* oncogene. The prognosis of disseminated neuroblastoma is better in infants (80% 5-year survival) when compared to children above one year of age (45% 5-year survival). Survival and outcomes have improved substantially over the last 30 years with multimodality modern therapy, including chemotherapy, radiation therapy, surgery, myeloablative therapy with stem cell transplant, immunotherapy, and differentiation therapy [46, 52]. In addition, tumor cell vaccination and immunotherapy treatments are being tested in phase I/II clinical trials. 

### 3.3. Optic Pathway Glioma

Optic pathway gliomas can involve any portion of the visual pathway, including the hypothalamus, optic disc, nerve, chiasm, geniculate nucleus, and optic radiations. Categorized as juvenile pilocytic astrocytomas, they account for 4–6% of all brain tumors in children, and the median age of diagnosis is 5–9 years [5, 53, 54]. Approximately half of all patients with optic pathway gliomas have neurofibromatosis (NF1), an autosomal dominant mutation. Tumor incidence among patients with NF1 ranges from 30 to 58%, and symptomatic lesions only occur in 1–5% of patients [5]. Bilateral involvement of the optic pathways is virtually pathognomonic for NF1. Histology typically shows low-grade gliomas (WHO grade I-II); however, individual tumors can display a wide spectrum of disease progression [56]. 

The vast majority of optic pathway gliomas is slow growing and can go unrecognized clinically for a long time. Patients typically present with decreased visual acuity, which may worsen with growth of the glioma within the optic pathway nerves. Other signs include optic disc edema, pallor, atrophy, relative afferent pupillary defect, decreased color vision, pupil dysfunction, visual field defects, ocular motility problems, and proptosis. If the tumor arises within the hypothalamus, endocrinopathies such as accelerated growth and precocious puberty may manifest [5]. Hypothalamic lesions can cause hydrocephalus, and 15-year mortality rates approach 50% [57].

**Figure 6:**
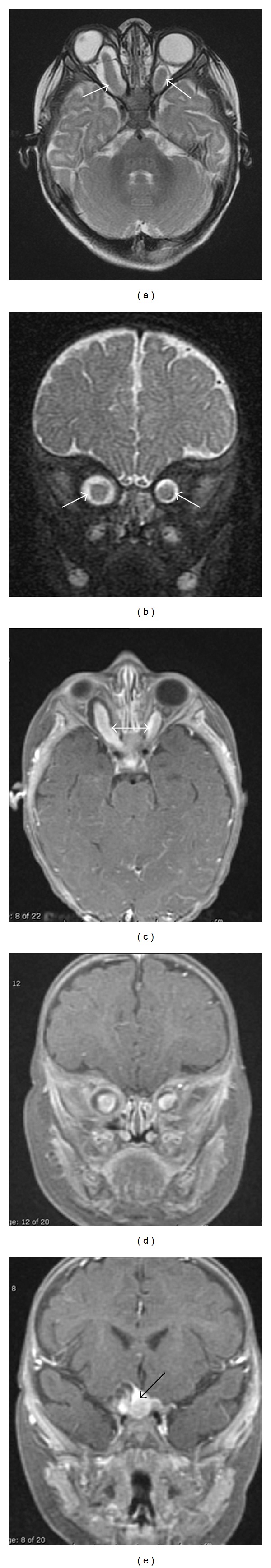
5 months old with eye deviation. Axial T2-weighted (a) and coronal T2-weighted fat-saturated (b) images demonstrate fusiform enlargement of the bilateral optic nerves, right greater than left (white arrows), with associated deviation of the right globe. Postcontrast axial (c) and coronal ((d), (e)) T1WI with fat saturation demonstrate diffuse enhancement of the optic nerves (double white arrow) with extension into the optic chiasm (black arrow). This is compatible with bilateral optic nerve gliomas and is virtually diagnostic of neurofibromatosis type 1.

The appearance of optic pathway gliomas on CT and MR is characteristic and specific for the disease process; however, MR is more sensitive for detection of smaller lesions. The gliomas typically show fusiform enlargement of the optic nerve (Figure 6). Additional features include widening of the optic canal, variable contrast enhancement, and rarely eccentric enlargement of the nerve and cystic degeneration [5]. MR imaging evaluates the intracranial disease better than CT and avoids radiation. Tumors typically demonstrate iso- to hypointense signal on T1WI, hyperintense signal on T2WI, and variable enhancement on postcontrast images. Posterior extension of the tumor can be seen as increased signal on post-contrast images and T2WI/FLAIR hyperintensity, which would influence treatment options. Optic pathway gliomas can be differentiated from optic nerve meningiomas, as meningiomas are dark on T2WI, originate from the meninges, and avidly enhance on post-contrast images [5].

Advanced MR techniques including magnetic resonance diffusion tensor imaging (MRDTI), diffusion-weighted imaging (DWI), and dynamic contrast enhancement (DCE) have been employed to further evaluate optic pathway gliomas. An early study in 2008 used DW and DCE imaging to calculate the diffusivity and permeability of optic pathway gliomas and found that clinically aggressive gliomas had significantly higher permeability than clinically stable gliomas and may warrant closer followup [58]. A separate group found that MRDTI may offer increased sensitivity over conventional imaging in identifying abnormalities in the optic pathways of patients with NF1 and may be able to quantitatively assess the extent of white matter disease [59]. Further long-term data will be needed to determine whether these newer techniques will prove clinically useful in the general population.

Some gliomas enter a stage of stable or slow growth, and some have even spontaneously improved. Tumors confined to the optic nerve at presentation rarely extend into the chiasm or develop extradural extension or metastases. Given the slow growing nature of most of these tumors, active surveillance can be a reasonable option. In patients with progressive disease, chemotherapy is the mainstay of treatment, usually with carboplatin and vincristine. Radiation therapy with fractionated gamma knife radiosurgery can be used after chemotherapy failure and retards or reverses progress in many cases. However, long term-data does not suggest that this specifically reduces mortality or vision loss [60]. Radiation treatment also exposes the patient to the potential side effects of radiation treatment, including dry eye, neovascular glaucoma, cataract, retinopathy, and optic neuropathy. Studies to optimize the treatment protocols for these patients have not been done. Surgery is also a therapeutic option, especially in patients with aggressive disease and no vision in the affected eye. A combined transcranial and orbital approach for en bloc resection of optic nerve gliomas with preservation of the annulus of Zinn has been described to be effective for debulking a proptotic blind eye [61]. The five-year overall survival was 96% and 20% for patients with low- and high-grade optic nerve gliomas, and these findings correlated with the tumor grade and not with age at diagnosis, receipt of radiation therapy, or extent of surgical resection [62].

### 3.4. Plexiform Neurofibroma

Plexiform neurofibroma (PNF) is a hamartoma of neuroectodermal origin representing 1-2% of all orbital tumors and typically arises in the first decade of life [63]. Presence of a PNF is diagnostic of NF1. The lesion can involve any peripheral nerve but usually involves sensory nerves in the orbit or eyelid and can cause widening of the superior orbital fissure as well as dysplasia of the greater sphenoid wing [63].

Eyelid PNFs have a characteristic S shape due to thickening, fat deposition, and horizontal redundancy. Orbital involvement can cause globe proptosis, bony expansion, and sphenoid dysplasia that can lead to temporal lobe herniation and pulsatile exophthalmos [57, 58]. Complete ptosis may result from increasing bulk of the upper lid. The lesions feel soft and have been described as feeling like “a bag of worms.” Irritation of the upper palpebral conjunctiva rubbing against the lower lashes can cause significant discomfort. Routine ophthalmologic examination is necessary, as PNFs can lead to amblyopia from occlusion, anisometropia, and strabismus [64]. Risk of malignant transformation to a sarcoma can be found in up to 7–10% of PNFs, and risk of ipsilateral glaucoma can be seen in up to 50% of patients with PNFs [58, 59]. 

**Figure 7:**
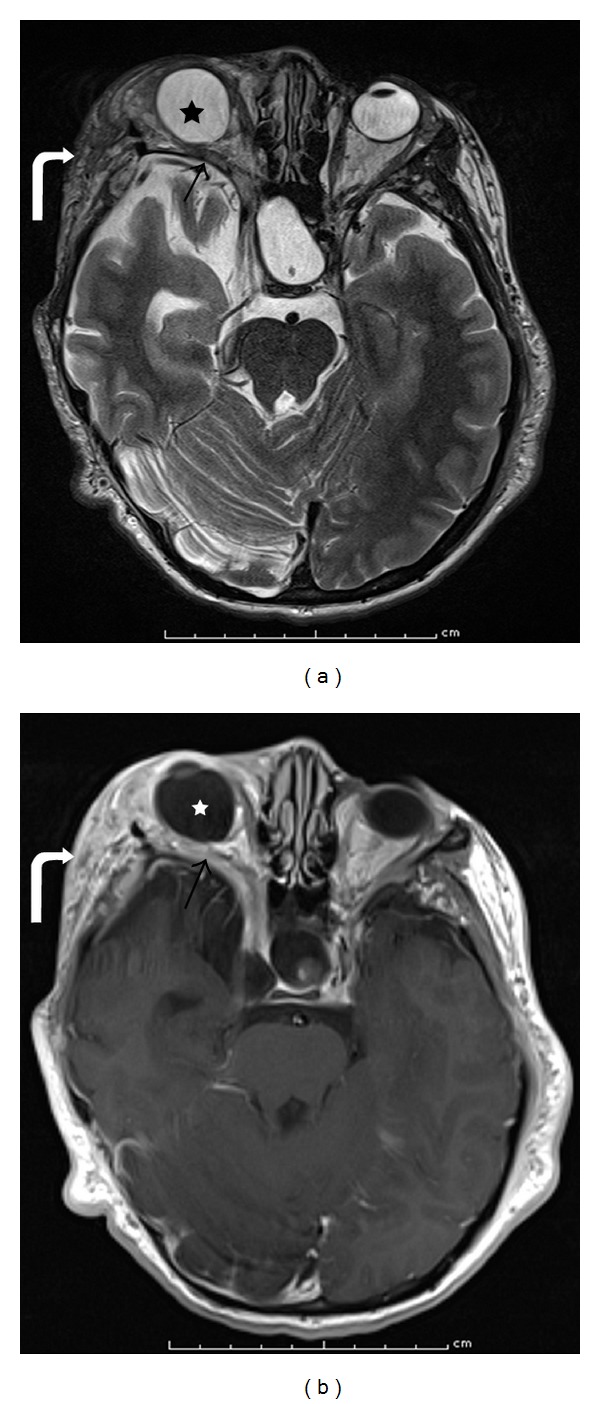
11 years old with right pulsatile proptosis. MR axial T2-weighted image (a) and T1-weighted postcontrast image (b) showing sphenoid wing dysplasia with mild temporal lobe herniation (arrow); plexiform neurofibroma with areas of T2 hyperintensity, heterogeneous enhancement, and extension lateral to the orbit into the infratemporal fossa (curved arrow); and buphthalmos and exophthalmos (star) of the right orbit.

Imaging with CT or MR can help determine extent of tumor involvement, amount of bony dysplasia, and can aid in surgical planning. PNFs appear as diffuse, irregular masses that cross multiple tissue planes with other features including thickening of the soft tissues, irregular intraconal fat, irregular nodularity of the optic nerve sheath, and thickening of the sclera/choroid [63]. Tumor can infiltrate into the orbit with involvement of the sensory nerves, lacrimal gland, and extraocular muscles. Bony involvement can lead to varying degrees of sphenoid dysplasia, expansion of the middle cranial fossa, expansion of the anterior orbital rim, and expanded orbital foramina secondary to trigeminal nerve involvement [65] with resultant exophthalmos and buphthalmos. MRI has improved soft-tissue resolution over CT and may allow improved visualization of tumor extension into the adjacent soft-tissues (Figure 7). The tumor appears hypointense on T1WI, hyperintense on T2WI, and has variable enhancement on postcontrast images. Extent of brain herniation through defects in the sphenoid can be better demonstrated on MRI.

PNFs show considerable enlargement during childhood and adolescence and can result in severe disfigurement. Treatment is directed at the relief of specific symptoms. Generally, complete surgical excision of the plexiform neurofibroma is not possible. Surgical debulking and frontalis suspension procedures can reduce the ptosis and allow for binocular vision. However, the condition is often progressive. Chronically inflamed conjunctiva occasionally requires resection.

### 3.5. Leukemia

Leukemia is the most common malignancy of childhood in the USA, with acute lymphoblastic leukemia accounting for 80% of all cases and acute myeloid leukemia (AML) accounting for 20% of cases [66, 67]. In patients with AML, a rare solid tumor made of primitive granulocyte precursors may form within the orbit and is called a granulocytic sarcoma [53]. This mass may present prior to or after the diagnosis of AML and can be a sign of relapse in treated patients [49, 61]. Mean age of presentation is around 8-9 years with unilateral disease in 90% of cases [63].

The most common manifestation is leukemic retinopathy, which presents with flame-shaped retinal hemorrhages, sometimes with white centers, in the nerve fiber layer. Additionally, perivascular infiltrations, microinfarctions, and discrete tumor infiltrations can be seen. Central scotomas and serous retinal detachments of the macula have also been reported. Anterior chamber hyphemas, pseudohypopyons, and iris masses can also be presenting signs of intraocular leukemia. Optic nerve infiltration needs to be distinguished from papilledema secondary to CNS leukemia. The less common orbital deposition of leukemic cells can present as a rapidly enlarging orbital mass, which can cause pain, eyelid swelling, ecchymosis, diplopia, and proptosis [49, 57]. This may be difficult to distinguish clinically from orbital cellulitis. Enhancing depth imaging optical coherence tomography can be useful in quantifying the increase in choroidal thickness, which can improve after systemic chemotherapy [68].

Granulocytic sarcomas are somewhat irregular homogenous masses with lateral orbital predilection that tend to encase rather than invade the adjacent lacrimal gland and extraocular muscles [53]. CT may demonstrate bony erosion and subperiosteal reaction. On MR, the mass is iso- to hypointense to muscle on T1-weighted sequences, heterogeneously iso- to hyperintense on T2-weighted sequences, and has homogenous enhancement on postcontrast sequences [53].

Treatment, usually systemic chemotherapy and bone marrow transplantation, is guided by the pediatric oncologist and customized based on specific genetic features of the malignancy. A leukemic infiltrate of the optic nerve can cause optic nerve elevation, and this is a medical emergency because of potential permanent loss of central vision when left untreated. The treatment is low-dose radiation therapy often combined with intrathecal chemotherapy as soon as possible. 

### 3.6. Lymphoproliferative Disease and Orbital Inflammatory Syndrome (OIS)

Lymphoproliferative disease is rare in children, representing less than 5–10% of orbital lesions. Lymphoid hyperplasia accounts for 10–40%, and non-Hodgkin's lymphoma accounts for 60–90% of the lesions. Lymphoproliferative disease can be categorized into reactive lymphoid hyperplasia, atypical lymphoid hyperplasia, and ocular adnexal lymphoma. Isolated orbital lymphoma is usually a low grade B-cell lymphoma, a subtype of non-Hodgkin's lymphoma. Diffuse large B-cell lymphomas are usually systemic.

These lesions are most commonly seen in the superotemporal quadrant with a predilection for the lacrimal gland; however, they may present with diffuse extraocular muscle involvement that can mimic thyroid ophthalmopathy [66]. Patients typically present with gradual, painless progression of proptosis. Patients with orbital lymphoproliferative lesions must have a systemic evaluation for lymphoma. In followup six months after presentation with orbital lymphoproliferative tumor, 16% of patients will develop systemic lymphoma [69]. OIS often involves the lacrimal gland and/or extraocular muscles and can often mimic lymphoproliferative disease with proptosis. 

**Figure 8:**
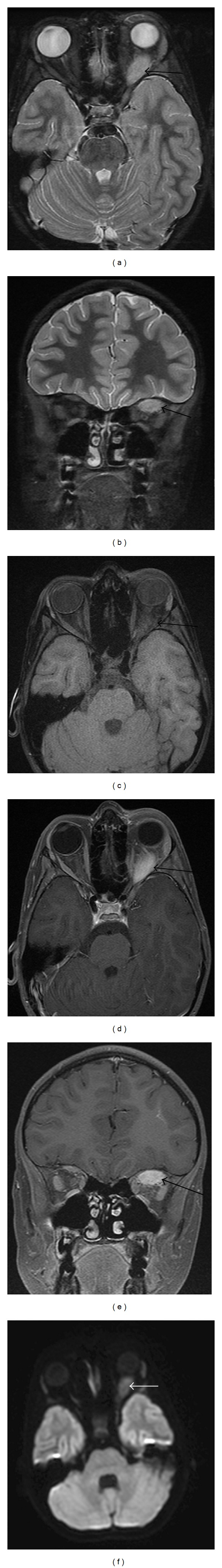
13 years old with Crohn disease. MR axial (a) and coronal (b) T2-weighted fat saturated, MR precontrast axial T1-weighted fat saturated (c), postcontrast axial (d) and coronal (e) T1-weighted fat saturated, and axial diffusion-weighted (f) images showing an infiltrative, diffusely enhancing soft-tissue mass involving the left superior rectus muscle (black arrows) with restricted diffusion (white arrow). This lesion responded to steroid therapy, supporting the suspected diagnosis of OIS. The fact that this lesion showed intermediate diffusion restriction (ADC value of 0.9-1 × 10^−3 ^mm^2^/s) demonstrates the difficulty in discriminating between lymphoproliferative disease and OIS.

**Figure 9:**
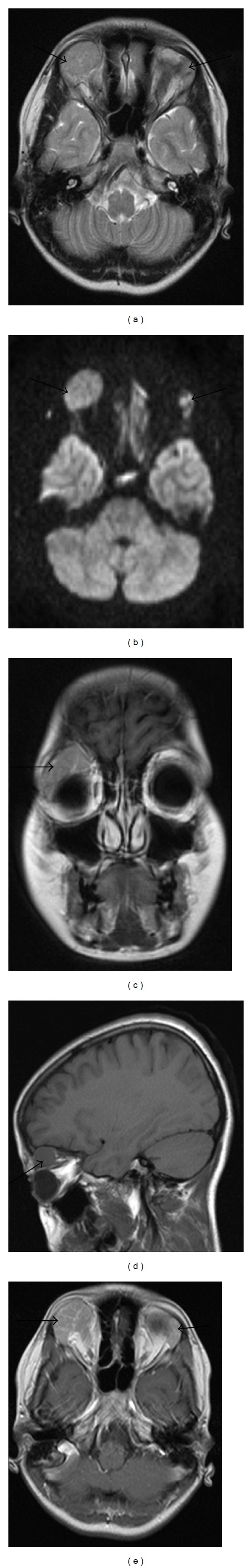
5 years old with history of combined variable immune deficiency. Axial T2-weighted image through the superior orbit (a) shows lobulated, isointense soft-tissue masses in the bilateral lacrimal glands (arrows). On diffusion-weighted images (b), the masses show restricted diffusion, corroborated by ADC maps (not shown). Precontrast coronal (c) and sagittal (d) T1WI show the well-circumscribed, mildly hypoattenuating mass centered in the right lacrimal gland that homogenously enhances on postcontrast T1-weighted image (e). Biopsy confirmed lymphoid hyperplasia bilaterally.

**Figure 10:**
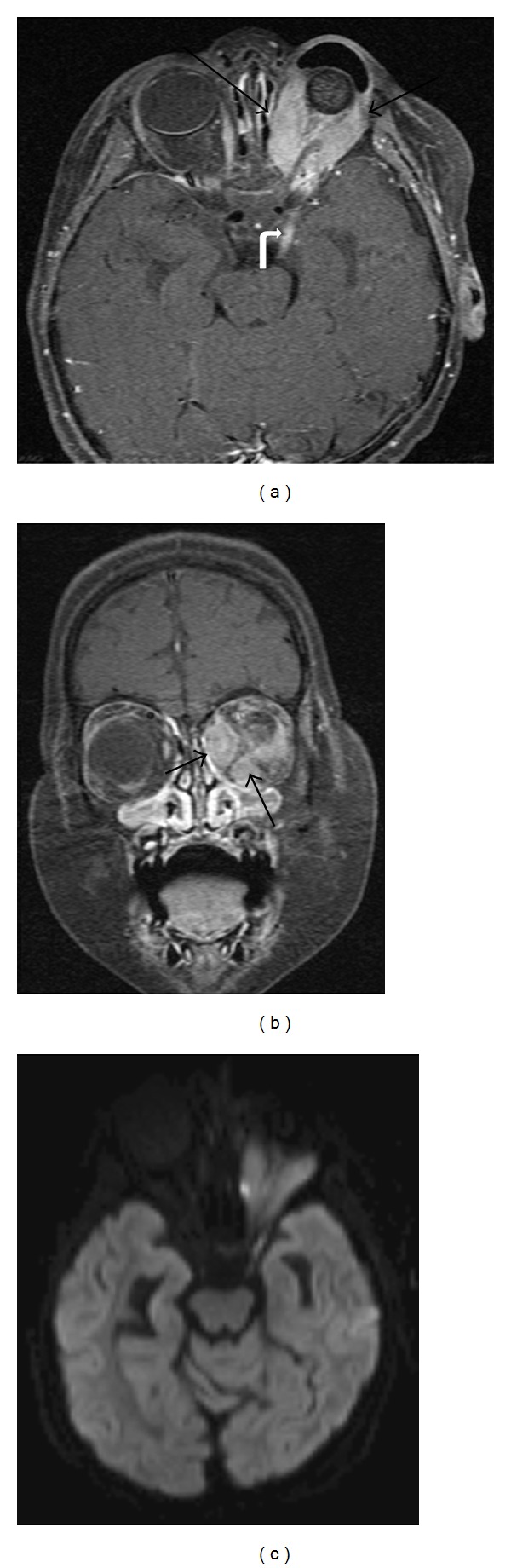
8 years old with orbital prosthesis status postenucleation for retinoblastoma presents with proptosis. MR axial (a) and sagittal (b) T1-weighted postcontrast fat-saturated images demonstrate homogeneously enhancing infiltrative tissue behind the prosthesis (black arrows). Increased signal along the course of the optic nerve is consistent with perineural spread of disease (curved white arrow). Axial diffusion-weighted image at the same level shows increased signal within the mass due to highly cellular tissue (decreased diffusion, corroborated by ADC maps (not shown)). Biopsy confirmed lymphoma.

Differentiating lymphoproliferative disease from OIS is difficult on CT and MR, as both diseases can show enlargement of the lacrimal glands or extraocular muscles. MR is more sensitive in discriminating between lymphoid disease and OIS, as lymphoproliferative disease has been shown to have significantly more restricted diffusion than OIS (Figure 8) [65, 70]. Lymphomas typically have ADC values less than 1.0 × 10^−3 ^mm^2^/sec, and OIS cases typically have ADC values greater than 1.0 × 10^−3 ^mm^2^/sec; however, overlap does exist. Lymphoid hyperplasia and lymphoma have overlapping imaging characteristics as well. Hyperplasia (Figure 9) may appear more circumscribed, and lymphoma (Figure 10) may appear more infiltrative [71].

OIS is often painful in nature due to acute inflammation and is treated with corticosteroids [72]. Lymphoproliferative lesions confined to the orbit are treated with radiation. Proper handling of the specimen is critical in appropriate tissue processing and interpretation of findings by the ophthalmic pathologist. Ultimately, a combination of clinical, radiologic, and histologic evaluation will have the best chance of correctly diagnosing this rare pediatric disease. 

### 3.7. Dermoid and Epidermoid Inclusion Cysts

Cystic structures are the most common orbital lesions in the pediatric population. Orbital cysts are either congenital or acquired but are mostly benign. Dermoid cysts are the most common orbital cystic tumor of childhood and arise from ectoderm trapped in bony sutures during embryogenic migration or from failure of surface ectoderm to separate from the neural tube. Epidermoid cysts present similarly but lack the dermal elements in the cyst wall [49, 57]. Dermoid cysts can occur bilaterally.

**Figure 11:**
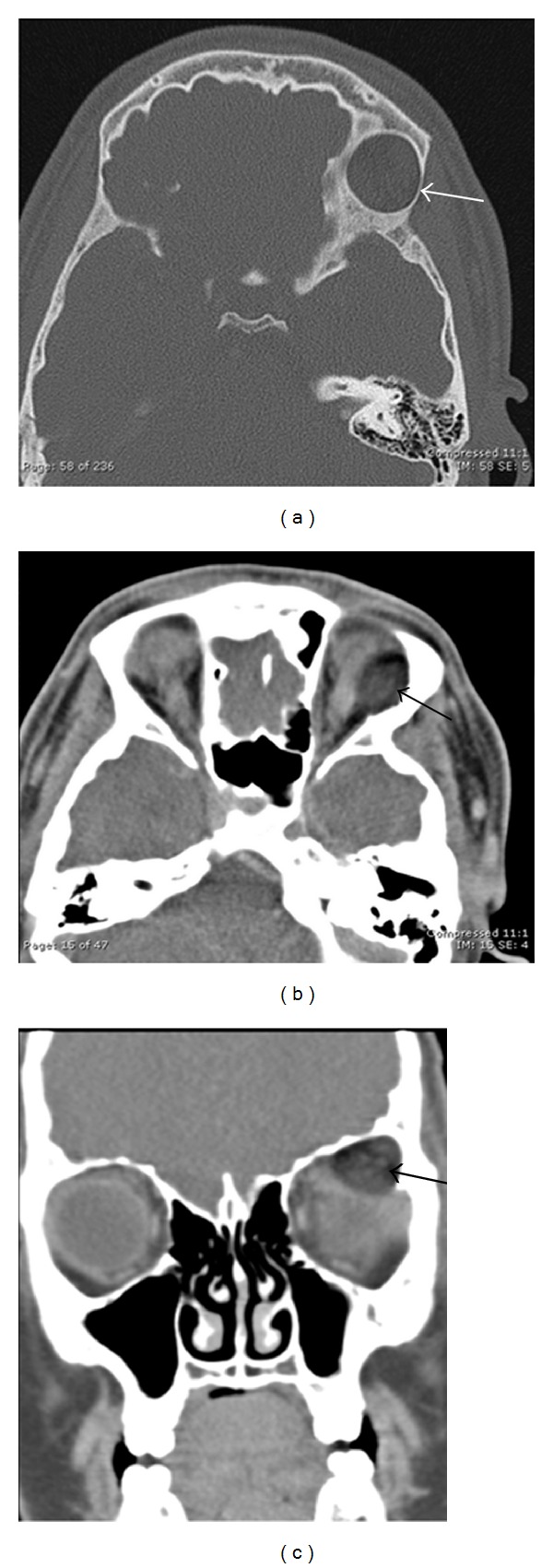
18 years old with diplopia. Axial CT with bone algorithm (a) shows a well-circumscribed lesion in the superolateral left orbit that causes adjacent bone thinning (white arrow) and sclerosis. Axial (b) and coronal (c) CT with soft-tissue algorithm show low attenuation fat within the mass (black arrows), diagnostic of a dermoid inclusion cyst.

Most dermoids and epidermoids present in the childhood and teen years as a slow growing, painless, subcutaneous mass near the superotemporal orbit and frontozygomatic suture [49, 57]. When growth is outward into the eyelid, cysts present in early childhood, and when they grow inward into the orbit they present later in life. The mass is usually nontender, slightly fluctuant to firm, and mimics a lacrimal gland tumor. Occasionally, the globe may be minimally displaced, but vision is rarely affected. Deep orbital or intraconal dermoid cysts may present with proptosis, ocular motility disturbances, and orbital nerve compression. The inclusion cysts may rupture spontaneously or with trauma, resulting in an intense inflammatory response in the surrounding tissues that may mimic an orbital cellulitis [53]. Additionally, orbital fistulas or sinus tracts can be contiguous with the dermoid and also result in orbital cellulitis, which may be the presenting sign of an orbital dermoid [73]. Both dermoid and epidermoid cysts cause adjacent bony remodeling secondary to their slow growth, which is best seen on CT as an adjacent rim of dense bone (Figure 11). Superficial dermoids can be evaluated with US and demonstrate smooth contours, variable echogenicity, and no demonstrable internal vascularity [74].

**Figure 12:**
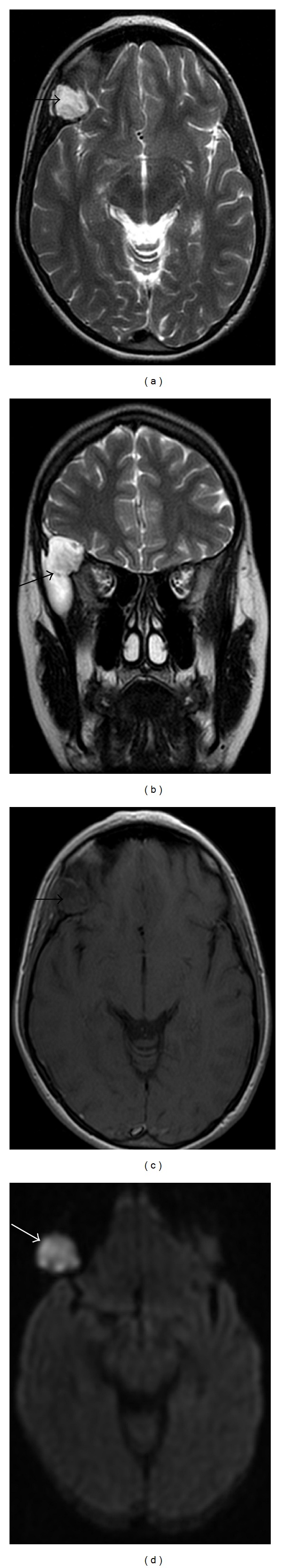
Patient with right orbital mass. MR axial (a) and coronal (b) T2-weighted, axial T1-weighted (c), and axial diffusion-weighted image (d) images show a T2 hyperintense, T1 isointense mass in the superolateral orbit, and sphenoid wing with restricted diffusion corroborated on ADC map (not shown), consistent with an epidermoid inclusion cyst.

Epidermoid cysts are well-circumscribed unilocular cysts that appear similar to cerebrospinal fluid on both MR and CT but can be clearly differentiated on MR DWI (Figure 12) where they appear high in signal intensity [53]. Dermoid inclusion cysts have a similar well-circumscribed margin and unilocular appearance; however, unlike epidermoid cysts, dermoids have characteristic CT attenuation closer to fat (Figure 11) with lipid signal on MR that suppresses on fat-saturated images [53]. This can be attributed to the sebaceous secretions within the dermoid inclusion cyst. Dermoid inclusion cysts also have a discernible wall that can enhance on post-contrast images and can contain dystrophic calcifications best seen on CT. 

Once diagnosed, management is based on the location of involvement and history of progression. If the lesion progresses and results in extraocular motor disturbances or compression of cranial nerves, this is an indication for treatment. Treatment is surgical en bloc resection of the lesion and any related sinus tracts. Traditional surgical approaches include incisions over the mass; above, below, or through the brow; parallel to superior orbital rim; via lateral canthotomy and lateral orbitotomy. The upper blepharoplasty approach [75] provides excellent scar camouflage and has been effective, especially in frontozygomatic dermoid cyst excision [76]. Deep orbital cysts are a challenge with a more difficult approach. Cases have been reported in which incomplete surgical resection resulted in malignant transformation to invasive squamous cell carcinoma [77].

### 3.8. Langerhans Cell Histiocytosis

Langerhans cell histiocytosis (LCH) is a disease characterized by abnormal proliferation of Langerhans cells and was previously thought to encompasses three clinical syndromes: Letterer-Siwe disease, which involves the vital organs of infants with often fatal outcomes; Hand-Schuller-Christian disease, seen in young children with diabetes insipidus, osteolytic calvarial defects, and exophthalmos; and eosinophilic granuloma, an indolent disease limited to the bones of older children or adults [49, 71]. LCH is now thought to represent a spectrum of disease ranging from benign unifocal bone disease to more aggressive multisystem disease [78]. Eosinophilic granuloma, the most localized and benign form of LCH, usually presents in children less than 4 years of age as unifocal bone disease with possible orbital involvement. LCH has been reported to have a higher male predominance, commonly involves the superotemporal orbit, and manifests with proptosis, ptosis, erythema, and enlarging palpebral fissures. 

**Figure 13:**
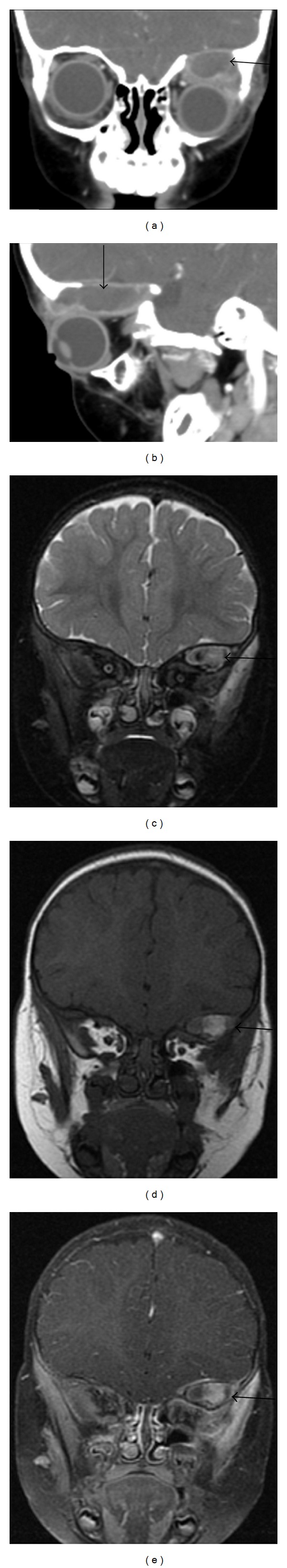
4 years old with known Langerhans cell histiocytosis. Coronal (a) and sagittal (b) contrast-enhanced CT images demonstrate a well-defined, osteolytic mass in the left orbital roof with rim enhancement. MR coronal T2-weighted fat-saturated (c), T1-weighted (d), and T1-weighted postcontrast fat-saturated (e) images show heterogeneous signal with possible enhancement in the left orbital roof mass with displacement of the globe inferiorly (not shown).

Imaging can help define the extent of disease and osseous destruction. CT and MRI provide more information than ultrasound. On CT, LCH manifests as a soft-tissue lesion that replaces/destroys osseous structures, can be well-defined or diffusely homogeneous, and shows moderate to marked enhancement after contrast administration (Figures 13(a) and 13(b)). The soft-tissue mass may extend into the orbit, temporal fossa, forehead, face, and epidural space [53]. MR should be used to better evaluate intracranial extension of disease. The mass replaces the normal bright signal of marrow with heterogeneous signal on T1WI, can be hyperintense or hypointense on T2WI, and demonstrates enhancement on T1-weighted post-contrast images (Figures 13(c), 13(d), and 13(e)) [53]. If there is clinical concern for pituitary involvement, the pituitary stalk and gland can be evaluated with a concurrent brain MRI to evaluate for abnormal thickening/enhancement or absence of the posterior pituitary bright spot [79]. Technetium-99m bone scintigraphy may demonstrate either increased or decreased radiotracer uptake in lesions. If multifocal bone disease is suspected, technetium-99m bone scintigraphy, or fluorine-18 fluorodeoxyglucose PET/CT, in conjunction with radiographic skeletal survey, may help identify more remote extraorbital lesions that require attention.

Isolated asymptomatic lesions may be observed. Painful lesions with favorable access can be excised or directly injected with steroids. Patients can be medically managed with systemic corticosteroids or chemotherapy, depending on the severity of the disease and location of involvement. Lesions are also treated with low-dose radiation. These patients must be followed because they can develop juxtaneural or intracranial extension and can progress to multifocal disease [49, 72].

## 4. Vascular Lesions

### 4.1. Infantile Hemangioma

Infantile hemangiomas are hamartomas of capillary endothelial cells. They are the most common vascular tumor of childhood in the orbit and are classified into preseptal, intraorbital (involving the postseptal orbit), and compound/mixed, involving both [63]. 

Most cases are diagnosed within the first weeks to months of life, after which the hemangioma begins a proliferative phase for up to 10 months. During this phase, the lesion may be complicated by hemorrhage, ulceration, cause amblyopia by obstructing the visual axis or inducing astigmatism, cause glaucoma by compression of the ocular outflow channels, stretch the optic nerve, and cause corneal ulceration [37]. After the first year of life, the lesion stabilizes and follows a course of prolonged involution for up to 10 years [36, 57]. The number and size of vessels decrease during the involutional phase [36, 74]. Hemangiomas vary widely in size from clinically insignificant to large disfiguring masses. When the hemangioma is superficial within the skin, it often has a characteristic strawberry color with deeper lesions appearing bluish. Intraorbital hemangiomas can present with proptosis, and further imaging must be performed for full evaluation. Multifocal hemangiomas can be seen in up to a third of patients with involvement of the viscera and skin [80]. 

Imaging is indicated to assess for the depth of the lesion and its relationship with adjacent structures for any lesions with atypical features on physical exam or presentation, or an associated underlying syndrome. US is useful as an inexpensive initial exam and typically demonstrates a well-circumscribed mass with a vessel density of greater than 5 vessels/cm^2^ with variable echogenicity. Spectral waveforms show a high-flow system with low resistance; however, no arteriovenous shunting should be identified. 

**Figure 14:**
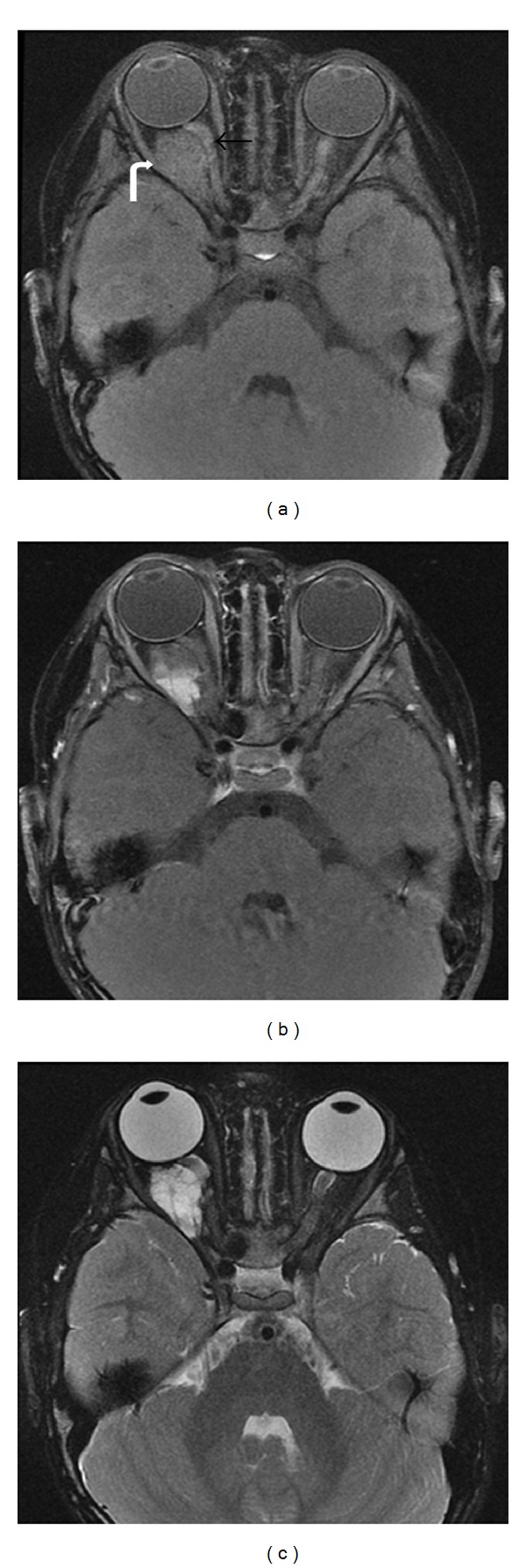
Patient with blue rubber bleb nevus presenting with right proptosis. Precontrast T1-weighted axial images with fat saturation (a) show a well-defined, lobulated, and intraconal mass (curved arrow) displacing the right optic nerve (black arrow). Postcontrast T1-weighted fat-saturated axial image (b) shows patchy, irregular, and enhancement. On T2-weighted fat-saturated image (c), the mass is hyperintense with linear low-intensity areas, likely flow voids. These findings were consistent with a hemangioma.

If further characterization is needed, MRI offers more sensitive evaluation of deeper lesions and their relationship to adjacent structures while avoiding the ionizing radiation of CT. Additionally, MR angiography (MRA) can be used to assess flow characteristics within the vascular lesions. Typical hemangiomas are well-circumscribed solid masses with arterial flow voids, intermediate signal on T1WI, increased signal on T2WI, marked enhancement on post-contrast images, and high flow on time-resolved MRA (Figure 14). During the involutional phase, the flow voids and enhancement decrease, and fibrofatty deposition can be identified as areas of increased signal on T1WI [36, 74]. CT findings are similar to MRI with decreased soft tissue and may be useful in patients who cannot tolerate sedation or have other contraindications to MRI.

If complications of the capillary hemangioma arise, intralesional corticosteroid injections into the eyelid can be safely performed but should be done with caution. Injection pressures routinely exceed the systolic blood pressure during these procedures [81], making the complication of retrograde embolization possible. Central retinal artery occlusion (CRAO) is a documented complication of steroid injection treatment for eyelid hemangioma. Prolonged adrenal suppression after an injection of triamcinolone/betamethasone has been reported in several patients [82]. Additionally, intravenous propranolol [83], oral propranolol [84] have also been shown to decrease the size of non vision-threatening lesions. Currently, twice daily topical 0.25% timolol maleate gel is used, as it is successful in treating eyelid hemangiomas [85].

### 4.2. Venous-Lymphatic Malformations or Lymphaticovenous Malformations

Venous-lymphatic malformations (VLMs) encompass a spectrum of complex congenital lesions of the orbit that are the result of maldevelopment of lymphatic and vascular structures during embryologic life. VLMs are composed of a mixture of venous and lymphatic vessels that vary in composition based on location. Deep lesions tend to be more venous in nature, while superficial lesions contain more lymphatic components. This corresponds to the normal vessel and lymphatic distribution of the orbit. They account for 4% of all expanding pediatric orbital masses and are usually evident by two years of age [37].

Periorbital malformations may cause major morbidity, including intralesional bleeding, intermittent swelling, blepharoptosis, fluctuating proptosis, pain, amblyopia, chemosis, astigmatism, and strabismus. Forty percent of children have diminished vision in the affected eye [86]. Sequelae include exposure keratopathy and compressive optic neuropathy. Superficial lesions are identified earlier and can extend to the forehead and cheek; deeper lesions present later with restricted ocular motility, acute proptosis secondary to hemorrhage, or acute enlargement with concomitant upper respiratory tract infection [37]. When hemorrhage occurs, variable-sized chocolate colored cysts may be identified [87]. Hormonal changes associated with puberty or pregnancy can cause accelerated growth [88]. Presentation due to slowly progressive enlargement is less common.

**Figure 15:**
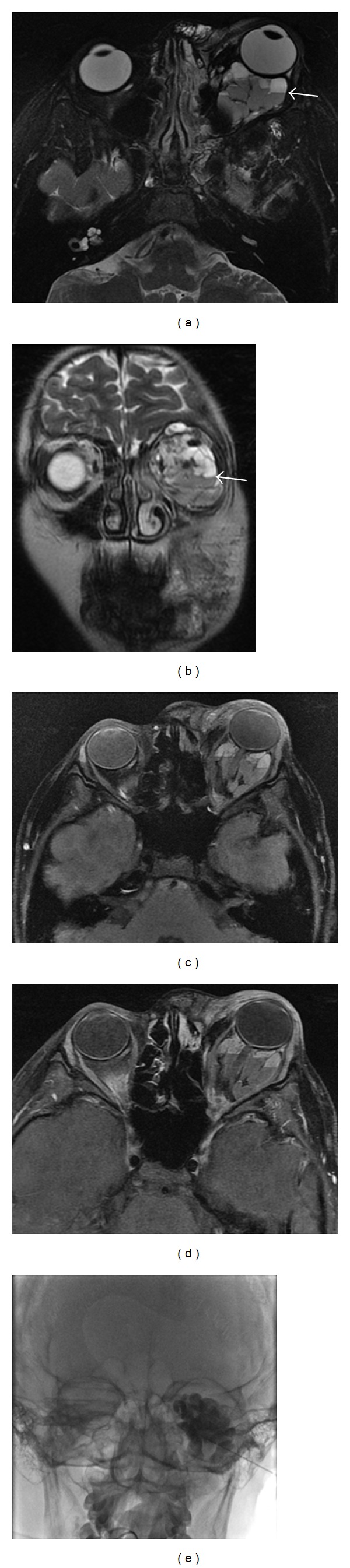
17 years old with acute worsening of left eye proptosis secondary to intralesional hemorrhage. MR axial T2-weighted fat-saturated (a) and coronal T2WI (b), axial T1-weighted fat-saturated image (c), and postcontrast T1-weighted fat-saturated image (d) showing a lobulated, trans-spatial mass with fluid-fluid levels, and trace rim enhancement (white arrows). The fluid-fluid levels are due to intralesional hemorrhage and are almost pathognomonic for a VLM. The lesion was treated with sclerotherapy using bleomycin and sodium tetradecyl sulfate (e).

The imaging characteristics of VLMs reflect their gross appearance with the mass appearing irregular, lobulated, infiltrating with ill-defined margins (due to lack of a capsule), and involving both the pre- and postseptal as well as the intra- and extraconal portions of the orbit [36, 80]. Macrocysts within the lesion can measure up to 2 cm with microcysts appearing solid [37]. MR imaging best evaluates the various components of the malformation with lymphatic/proteinaceous fluid best seen on T1WI, blood products best seen on T1 fat-suppressed images, and nonhemorrhagic fluid best seen on T2WI [88]. Various ages of hemorrhage within the cysts produce fluid-fluid levels that are nearly pathognomonic for the diagnosis of a VLM. A lack of flow voids helps distinguish this lesion from hemangiomas (Figure 15). CT can better delineate any associated bony changes of the orbit including widening of the orbital fissures and remodeling of the orbital walls but is less accurate than MRI in delineating the anatomic location and characterizing the individual components of the lesion [37]. Phleboliths can also be seen better on CT. US is limited in evaluation of the deeper structures, necessitating MR or CT. VLMs are associated with intracranial vascular anomalies in up to 70% of patients, and imaging of the brain should be performed concurrently [89]. 

Treatment of periorbital VLMs requires an interdisciplinary team that includes interventional and diagnostic radiologists, craniofacial surgeons, and ophthalmologists. Several interventions are usually required. Percutaneous sclerotherapy with 3% sodium tetradecyl sulphate (STD) [90] is an effective first-line therapy for macrocystic and microcystic VLM with negligible recurrence rates and improvement in vision and proptosis after treatment. Percutaneous sclerotherapy can also be used for recurrence after surgical intervention [91]. These lesions have also been historically treated with intralesional injections of OK-432 [92], a lyophilized mixture of group A *Streptococcus pyogenes*. This treatment, however, generates a significant local inflammatory reaction, which can include bleeding of the lesion and an increase in size [93], which resolves over weeks. There is also risk of vision loss from orbital compartment syndrome. Surgical management is often difficult and subtotal because of the diffuse infiltrating nature of the lesion [94]. A coronal approach is used for subtotal excision of frontotemporal-orbit malformations, but tarsal incisions are used for isolated eyelid lesions. Proptosis can be managed temporarily by tarsorrhaphy (suturing the eyelids partially closed) and definitively by expansion of the bony orbit. In some occasions, orbital exenteration is necessary [86].

## 5. Conclusion

A wide assortment of intraocular neoplasms and orbital masses involves the pediatric orbit and can present with various clinical manifestations. Given the complexity of their location and considerable morbidity that may be associated with biopsy at this site, a systematic and multidisciplinary approach is needed to help facilitate an accurate diagnosis. By understanding the clinical presentation and the characteristic imaging features of the disease, a narrow differential diagnosis can be formulated, and thus timely treatment can be initiated. The treatment of pediatric orbital and intraocular neoplasms, especially of retinoblastoma, has changed drastically over the last 10 years. During this same period, wider use of 3 T magnets with newer coils has improved signal quality of MR imaging, while new 3D pulse sequences now allow slice thicknesses of less than 1 mm. Furthermore, DWI imaging has recently emerged as an important diagnostic tool used to differentiate malignant and benign neoplasms. As imaging hardware and advanced imaging techniques become more refined, diagnostic accuracy should continue to improve. Advancements in drug delivery systems and therapies will hopefully translate into decreased morbidity and mortality in this young patient population. 

## Supplementary Material

Supplementary Table 1: The International Intraocular Retinoblastoma Classification system divides intraocular retinoblastomas into 5 groups (A through E), based on the chance that the eye can be salvaged using current treatments.

## References

[1] Kivela T (2009). The epidemiological challenge of the most frequent eye cancer: retinoblastoma, an issue of birth and death. *British Journal of Ophthalmology*.

[2] Dyer MA, Bremner R (2005). The search for the retinoblastoma cell of origin. *Nature Reviews Cancer*.

[3] Dimaras H, Kimani K, Dimba EA (2012). Retinoblastoma. *Lancet*.

[4] Dai S, Dimaras H, Héon E (2008). Trilateral retinoblastoma with pituitary-hypothalamic dysfunction. *Ophthalmic Genetics*.

[5] Chung EM, Specht CS, Schroeder JW (2007). From the archives of the AFIP: pediatric orbit tumors and tumorlike lesions: neuroepithelial lesions of the ocular globe and optic nerve. *Radiographics*.

[6] Chantada G, Doz F, Antoneli CBG (2006). A proposal for an international retinoblastoma staging system. *Pediatric Blood & Cancer*.

[7] Galluzzi P, Hadjistilianou T, Cerase A, De Francesco S, Toti P, Venturi C (2009). Is CT still useful in the study protocol of retinoblastoma?. *American Journal of Neuroradiology*.

[8] de Graaf P, Pouwels PJW, Rodjan F (2012). Single-shot turbo spin-echo diffusion-weighted imaging for retinoblastoma: initial experience. *American Journal of Neuroradiology*.

[9] de Graaf P, Göricke S, Rodjan F (2012). Guidelines for imaging retinoblastoma: imaging principles and MRI standardization. *Pediatric Radiology*.

[10] Brisse HJ, Lumbroso L, Fréneaux PC (2001). Sonographic, CT, and MR imaging findings in diffuse infiltrative retinoblastoma: report of two cases with histologic comparison. *American Journal of Neuroradiology*.

[11] Mohney BG, Robertson DM, Schomberg PJ, Hodge DO (1998). Second nonocular tumors in survivors of heritable retinoblastoma and prior radiation therapy. *American Journal of Ophthalmology*.

[12] Friedman DL, Himelstein B, Shields CL (2000). Chemoreduction and local ophthalmic therapy for intraocular retinoblastoma. *Journal of Clinical Oncology*.

[13] Brichard B, De Bruycker JJ, De Potter P, Neven B, Vermylen C, Cornu G (2002). Combined chemotherapy and local treatment in the management of intraocular retinoblastoma. *Medical and Pediatric Oncology*.

[14] Antoneli CBG, Ribeiro KCB, Steinhorst F, Novaes PERS, Chojniak MM, Malogolowkin M (2006). Treatment of retinoblastoma patients with chemoreduction plus local therapy: experience of the AC Camargo Hospital, Brazil. *Journal of Pediatric Hematology/Oncology*.

[15] Smith SJ, Pulido JS, Salomão DR, Smith BD, Mohney B (2012). Combined intravitreal and subconjunctival carboplatin for retinoblastoma with vitreous seeds. *The British Journal of Ophthalmology*.

[16] Krishnakumar S, Mohan A, Kandalam M, Ramkumar HL, Venkatesan N, Das RR (2008). SRPK1: a cisplatin sensitive protein expressed in retinoblastoma. *Pediatric Blood and Cancer*.

[17] Adithi M, Kandalam M, Ramkumar HL, Subramanian A, Venkatesan N, Krishnakumar S (2006). Retinoblastoma: expression of HLA-G. *Ocular Immunology and Inflammation*.

[18] Shields CL, Shields JA, Cater J, Othmane I, Singh AD, Micaily B (2001). Plaque radiotherapy for retinoblastoma: long-term tumor control and treatment complications in 208 tumors. *Ophthalmology*.

[19] Abramson DH, Dunkel IJ, Brodie SE, Marr B, Gobin YP (2010). Superselective ophthalmic artery chemotherapy as primary treatment for retinoblastoma (chemosurgery). *Ophthalmology*.

[20] Abramson DH, Dunkel IJ, Brodie SE, Kim JW, Gobin YP (2008). A phase I/II study of direct intraarterial (ophthalmic artery) chemotherapy with melphalan for intraocular retinoblastoma initial results. *Ophthalmology*.

[21] Marr BP, Brodie SE, Dunkel IJ, Gobin YP, Abramson DH (2012). Three-drug intra-arterial chemotherapy using simultaneous carboplatin, topotecan, and melphalan for intraocular retinoblastoma: preliminary results. *The British Journal of Ophthalmology*.

[22] Shields CL, Kaliki S, Shah SU (2012). Minimal exposure (one or two cycles) of intra-arterial chemotherapy in the management of retinoblastoma. *Ophthalmology*.

[23] Gobin YP, Rosenstein LM, Marr BP, Brodie SE, Abramson DH (2012). Radiation exposure during intra-arterial chemotherapy for retinoblastoma. *Archives of Ophthalmology*.

[24] Vijayakrishnan R, Shields CL, Ramasubramanian A, Emrich J, Rosenwasser R, Shields JA (2010). Irradiation toxic effects during intra-arterial chemotherapy for retinoblastoma: should we be concerned?. *Archives of Ophthalmology*.

[25] Saunders T, Margo CE (2012). Intraocular medulloepithelioma. *Archives of Pathology & Laboratory Medicine*.

[26] Shields JA, Eagle RC, Shields CL, De Potter P (1996). Congenital neoplasms of the nonpigmented ciliary epithelium (medulloepithelioma). *Ophthalmology*.

[27] Chua J, Muen WJ, Reddy A, Brookes J (2012). The masquerades of a childhood ciliary body medulloepithelioma: a case of chronic uveitis, cataract, and secondary glaucoma. *Case Reports in Ophthalmological Medicine*.

[28] Ayres B, Brasil OM, Klejnberg C, Moura LR, Fernandes BF, Burner MB (2006). Ciliary body medulloepithelioma: clinical, ultrasound biomicroscopic and histopathologic correlation. *Clinical and Experimental Ophthalmology*.

[29] Brennan RC, Wilson MW, Kaste S, Helton KJ, McCarville MB (2012). US and MRI of pediatric ocular masses with histopathological correlation. *Pediatric Radiology*.

[30] Müller K, Zwiener I, Welker H (2011). Curative treatment for central nervous system medulloepithelioma despite residual disease after resection. Report of two cases treated according to the GPHO Protocol HIT 2000 and review of the literature. *Strahlentherapie und Onkologie*.

[31] Lindegaard J, Heegaard S, Toft PB, Nysom K, Prause JU (2010). Malignant transformation of a medulloepithelioma of the optic nerve. *Orbit*.

[32] Martin K, Rossi V, Ferrucci S, Pian D (2010). Retinal astrocytic hamartoma. *Optometry*.

[33] Demirci H, Shields CL, Shields JA, Honavar SG (2002). Spontaneous disappearance of presumed retinal astrocytic hyperplasia. *Retina*.

[34] de Juan E, Green WR, Gupta PK, Barañano EC (1984). Vitreous seeding by retinal astrocytic hamartoma in a patient with tuberous sclerosis. *Retina*.

[35] Mennel S, Meyer CH, Peter S, Schmidt JC, Kroll P (2007). Current treatment modalities for exudative retinal hamartomas secondary to tuberous sclerosis: review of the literature. *Acta Ophthalmologica Scandinavica*.

[36] Huh WW, Mahajan A, Esmaeli B (2011). Ophthalmic oncology. *Ophthalmic Oncology*.

[37] Chung EM, Smirniotopoulos JG, Specht CS, Schroeder JW, Cube R (2007). From the archives of the AFIP: pediatric orbit tumors and tumorlike lesions: nonosseous lesions of the extraocular orbit. *Radiographics*.

[38] Bonnin N, Nezzar H, Viennet A (2010). Case report of a 2-year-old child with palpebral rhabdomyosarcoma. *Journal Francais d’Ophtalmologie*.

[39] Shields JA, Shields CL, Scartozzi R (2004). Survey of 1264 patients with orbital tumors and simulating lesions: the 2002 Montgomery Lecture, part 1. *Ophthalmology*.

[40] Freling NJM, Merks JHM, Saeed P (2010). Imaging findings in craniofacial childhood rhabdomyosarcoma. *Pediatric Radiology*.

[41] Völker T, Denecke T, Steffen I (2007). Positron emission tomography for staging of pediatric sarcoma patients: results of a prospective multicenter trial. *Journal of Clinical Oncology*.

[42] Tateishi U, Hosono A, Makimoto A (2009). Comparative study of FDG PET/CT and conventional imaging in the staging of rhabdomyosarcoma. *Annals of Nuclear Medicine*.

[43] Klem ML, Grewal RK, Wexler LH, Schöder H, Meyers PA, Wolden SL (2007). PET for staging in rhabdomyosarcoma: an evaluation of PET as an adjunct to current staging tools. *Journal of Pediatric Hematology/Oncology*.

[44] Kumar J, Seith A, Kumar A (2008). Whole-body MR imaging with the use of parallel imaging for detection of skeletal metastases in pediatric patients with small-cell neoplasms: comparison with skeletal scintigraphy and FDG PET/CT. *Pediatric Radiology*.

[45] Krohmer S, Sorge I, Krausse A (2010). Whole-body MRI for primary evaluation of malignant disease in children. *European Journal of Radiology*.

[46] Lin C, Luciani A, Itti E (2010). Whole-body diffusion-weighted magnetic resonance imaging with apparent diffusion coefficient mapping for staging patients with diffuse large B-cell lymphoma. *European Radiology*.

[47] Kojima Y, Hashimoto K, Ando M (2012). Comparison of dose intensity of vincristine, d-actinomycin, and cyclophosphamide chemotherapy for child and adult rhabdomyosarcoma: a retrospective analysis. *Cancer Chemotherapy and Pharmacology*.

[48] Breneman J, Meza J, Donaldson SS (2012). Local control with reduced-dose radiotherapy for low-risk rhabdomyosarcoma: a report from the Children’s Oncology Group D9602 study. *International Journal of Radiation Oncology, Biology, Physics*.

[49] Oberlin O, Rey A, Anderson J (2001). Treatment of orbital rhabdomyosarcoma: survival and late effects of treatment—results of an international workshop. *Journal of Clinical Oncology*.

[50] Gutierrez JC, Fischer AC, Sola JE, Perez EA, Koniaris LG (2007). Markedly improving survival of neuroblastoma: a 30-year analysis of 1,646 patients. *Pediatric Surgery International*.

[51] D’Ambrosio N, Lyo J, Young R, Haque S, Karimi S (2010). Common and unusual craniofacial manifestations of metastatic neuroblastoma. *Neuroradiology*.

[52] Günalp I, Gündüz K (1995). Metastatic orbital tumors. *Japanese Journal of Ophthalmology*.

[53] Chung EM, Murphey MD, Specht CS, Cube R, Smirniotopoulos JG (2008). From the archives of the AFIP pediatric orbit tumors and tumorlike lesions: osseous lesions of the orbit. *Radiographics*.

[54] Boubaker A, Bischof Delaloye A (2003). Nuclear medicine procedures and neuroblastoma in childhood: their value in the diagnosis, staging and assessment of response to therapy. *Quarterly Journal of Nuclear Medicine*.

[55] Pflüger T, Schmid I, Coppenrath E, Weiss M (2010). Modern nuclear medicine evaluation of neuroblastoma. *The Quarterly Journal of Nuclear Medicine and Molecular Imaging*.

[56] Alvord EC, Lofton S (1988). Gliomas of the optic nerve or chiasm. Outcome by patients’ age, tumor site, and treatment. *Journal of Neurosurgery*.

[57] Lee AG, Dutton JJ (1999). A practice pathway for the management of gliomas of the anterior visual pathway: an update and an evidence-based approach. *Neuro-Ophthalmology*.

[58] Jost SC, Ackerman JW, Garbow JR, Manwaring LP, Gutmann DH, McKinstry RC (2008). Diffusion-weighted and dynamic contrast-enhanced imaging as markers of clinical behavior in children with optic pathway glioma. *Pediatric Radiology*.

[59] Filippi CG, Bos A, Nickerson JP, Salmela MB, Koski CJ, Cauley KA (2011). Magnetic resonance diffusion tensor imaging (MRDTI) of the optic nerve and optic radiations at 3T in children with neurofibromatosis type I (NF-1). *Pediatric Radiology*.

[60] Kurt G, Tonge M, Börcek AO (2010). Fractionated gamma knife radiosurgery for optic nerve tumors: a technical report. *Turkish Neurosurgery*.

[61] Shriver EM, Ragheb J, Tse DT (2012). Combined transcranial-orbital approach for resection of optic nerve gliomas: a clinical and anatomical study. *Ophthalmic Plastic and Reconstructive Surgery*.

[62] Mishra MV, Andrews DW, Glass J (2012). Characterization and outcomes of optic nerve gliomas: a population-based analysis. *Journal of Neuro-Oncology*.

[63] Dutton JJ, Escaravage GK (2011). *Ophthalmic Oncology*.

[64] Slopis JM, Schiffman JS, Esmaeli B (2011). Ophthalmic oncology. *Ophthalmic Oncology*.

[65] Jacquemin C, Bosley TM, Svedberg H (2003). Orbit deformities in craniofacial neurofibromatosis type 1. *American Journal of Neuroradiology*.

[66] Valvassori GE, Sabnis SS, Mafee RF, Brown MS, Putterman A (1999). Imaging of orbital lymphoproliferative disorders. *Radiologic Clinics of North America*.

[67] SEER Cancer Statistics Review 1975–2009 (Vintage 2009 Populations). http://seer.cancer.gov/csr/1975_2009_pops09/browse_csr.php?section=28&page=sect_28_table.01.html.

[68] Bajenova NV, Vanderbeek BL, Johnson MW (2012). Change in choroidal thickness after chemotherapy in leukemic choroidopathy. *Retina*.

[69] Demirci H, Shields CL, Karatza EC, Shields JA (2008). Orbital lymphoproliferative tumors: analysis of clinical features and systemic involvement in 160 cases. *Ophthalmology*.

[70] Evans DGR, Baser ME, McGaughran J, Sharif S, Howard E, Moran A (2002). Malignant peripheral nerve sheath tumours in neurofibromatosis 1. *Journal of Medical Genetics*.

[71] Sepahdari AR, Aakalu VK, Setabutr P, Shiehmorteza M, Naheedy JH, Mafee MF (2010). Indeterminate orbital masses: restricted diffusion at MR imaging with echo-planar diffusion-weighted imaging predicts malignancy. *Radiology*.

[72] Hink EM, Durairaj V (2011). *Ophthalmic Oncology*.

[73] Wells TS, Harris GJ (2004). Orbital dermoid cyst and sinus tract presenting with acute infection. *Ophthalmic Plastic and Reconstructive Surgery*.

[74] Neudorfer M, Leibovitch I, Stolovitch C (2004). Intraorbital and periorbital tumors in children—value of ultrasound and color Doppler imaging in the differential diagnosis. *American Journal of Ophthalmology*.

[75] Nelson KE, Mishra A, Duncan C (2011). Upper blepharoplasty approach to frontozygomatic dermoid cysts. *The Journal of Craniofacial Surgery*.

[76] Ruszkowski A, Caouette-Laberge L, Bortoluzzi P, Egerszegi EP (2000). Superior eyelid incision: an alternative approach for frontozygomatic dermoid cyst excision. *Annals of Plastic Surgery*.

[77] Holds JB, Anderson RL, Mamalis N, Kincaid MC, Font RL (1993). Invasive squamous cell carcinoma arising from asymptomatic choristomatous cysts of the orbit: two cases and a review of the literature. *Ophthalmology*.

[78] Vosoghi H, Rodriguez-Galindo C, Wilson MW (2009). Orbital involvement in langerhans cell histiocytosis. *Ophthalmic Plastic and Reconstructive Surgery*.

[79] Barnes PD, Robson CD, Robertson RL, Young Poussaint T (1996). Pediatric orbital and visual pathway lesions. *Neuroimaging Clinics of North America*.

[80] Haik BG, Jakobiec FA, Ellsworth RM, Jones IS (1979). Capillary hemangioma of the lids and orbit: an analysis of the clinical features and therapeutic results in 101 cases. *Ophthalmology*.

[81] Egbert JE, Paul S, Engel WK, Summers CG (2001). High injection pressure during intralesional injection of corticosteroids into capillary hemangiomas. *Archives of Ophthalmology*.

[82] Morkane C, Gregory JW, Watts P, Warner JT (2011). Adrenal suppression following intralesional corticosteroids for periocular haemangiomas. *Archives of Disease in Childhood*.

[83] Léauté-Labrèze C, Dumas de la Roque E, Hubiche T, Boralevi F, Thambo J-B, Taïeb A (2008). Propranolol for severe hemangiomas of infancy. *The New England Journal of Medicine*.

[84] Fridman G, Grieser E, Hill R, Khuddus N, Bersani T, Slonim C (2011). Propranolol for the treatment of orbital infantile hemangiomas. *Ophthalmic Plastic and Reconstructive Surgery*.

[85] Chambers CB, Katowitz WR, Katowitz JA, Binenbaum G (2012). A controlled study of topical 0.25% timolol maleate gel for the treatment of cutaneous infantile capillary hemangiomas. *Ophthalmic Plastic and Reconstructive Surgery*.

[86] Greene AK, Burrows PE, Smith L, Mulliken JB (2005). Periorbital lymphatic malformation: clinical course and management in 42 patients. *Plastic and Reconstructive Surgery*.

[87] Smoker WRK, Gentry LR, Yee NK, Reede DL, Nerad JA (2008). Vascular lesions of the orbit: more than meets the eye. *Radiographics*.

[88] Dhellemmes P, Brevière GM, Degrugillier-Chopinet C, Vinchon M (2010). Vascular lesions of the orbit in children. *Neurochirurgie*.

[89] Bisdorff A, Mulliken JB, Carrico J, Robertson RL, Burrows PE (2007). Intracranial vascular anomalies in patients with periorbital lymphatic and lymphaticovenous malformations. *American Journal of Neuroradiology*.

[90] Kok K, McCafferty I, Monaghan A, Nishikawa H (2012). Percutaneous sclerotherapy of vascular malformations in children using sodium tetradecyl sulphate: the Birmingham experience. *Journal of Plastic, Reconstructive & Aesthetic Surgery*.

[91] Hill RH, Shiels WE, Foster JA (2012). Percutaneous drainage and ablation as first line therapy for macrocystic and microcystic orbital lymphatic malformations. *Ophthalmic Plastic and Reconstructive Surgery*.

[92] Suzuki Y, Obana A, Gohto Y, Miki T, Otuka H, Inoue Y (2000). Management of orbital lymphangioma using intralesional injection of OK-432. *British Journal of Ophthalmology*.

[93] Lanuza García A, Bañon Navarro R, Llorca Cardeñosa A, Delgado Navarro C (2012). Unsuccessful treatment with OK-432 picibanil for orbital lymphangioma. *Archivos de la Sociedad Española de Oftalmología*.

[94] Berthout A, Jacomet PV, Putterman M, Galatoire O, Morax S (2008). Surgical treatment of diffuse adult orbital lymphangioma: two case studies. *Journal Francais d’Ophtalmologie*.

