# Supplementary material for: A Clinical Update and Radiologic Review of Pediatric Orbital and Ocular Tumors
Source: J Oncol. 2013 Mar 12;2013:975908. doi: 10.1155/2013/975908 (PMC3610355; doi:10.1155/2013/975908)
Supplement: Supplementary file 1 — Supplementary Table 1: The International Intraocular Retinoblastoma Classification system divides intraocular retinoblastomas into 5 groups (A through E), based on the chance that the eye can be salvaged using current treatments. [file 975908.f1.pdf]

Supplemental Table 1

International Classification for Intraocular Retinoblastoma

Group	Simple Reference	Specifications
**A**	Small tumor	Lesion ≤ 3 mm
**B**	Large tumor<br>Macula<br>Juxtapapillary<br>Subretinal fluid	Lesion > 3 mm or<br>- Macular Rb location ≤ 3 mm to foveola<br>- Juxtapapillary Rb location ≤ 1.5 mm to disc<br>- Subretinal fluid ≤ 3 mm from margin
**C**	Focal seeds	Lesion with<br>- Subretinal seeds ≤ 3 mm from lesion<br>- Vitreous seeds ≤ 3 mm from lesion<br>- Subretinal and vitreous seeds ≤ 3 mm from lesion
**D**	Diffuse seeds	Lesion with<br>- Subretinal seeds > 3 mm<br>- Vitreous seeds > 3 mm<br>- Both subretinal and vitreous seeds > 3 mm
**E**	Extensive retinoblastoma	Large tumor burden with anatomic or functional destruction of the eye and/or:<br>- Neovascular glaucoma<br>- Opaque media from anterior chamber, vitreous, or subretinal hemorrhage<br>- Post-laminar optic nerve invasion, > 2 mm of choroidal invasion, scleral, orbital, or anterior chamber involvement<br>- Lens- tumor touch<br>- Diffuse infiltrating tumor<br>- Phthisis or pre-phthisis

Legend

Table 1. The International Intraocular Retinoblastoma Classification system divides intraocular retinoblastomas into 5 groups (A through E), based on the chance that the eye can be salvaged using current treatments.
